# Transcriptome Analyses Shed New Insights into Primary Metabolism and Regulation of *Blumeria graminis* f. sp. *tritici* during Conidiation

**DOI:** 10.3389/fpls.2017.01146

**Published:** 2017-06-30

**Authors:** Fan-Song Zeng, Fabrizio Menardo, Min-Feng Xue, Xue-Jiang Zhang, Shuang-Jun Gong, Li-Jun Yang, Wen-Qi Shi, Da-Zhao Yu

**Affiliations:** ^1^College of Life Science, Wuhan UniversityWuhan, China; ^2^Key Laboratory of Integrated Pest Management on Crops in Central China, Ministry of AgricultureWuhan, China; ^3^Institute of Plant Protection and Soil Science, Hubei Academy of Agricultural SciencesWuhan, China; ^4^Institute of Plant and Microbial Biology, University of ZürichZürich, Switzerland

**Keywords:** *Blumeria graminis* f. sp. *tritici*, conidiation, metabolism, regulation, RNA-seq

## Abstract

Conidia of the obligate biotrophic fungal pathogen *Blumeria graminis* f. sp. *tritici* (*Bgt*) play a vital role in its survival and rapid dispersal. However, little is known about the genetic basis for its asexual reproduction. To uncover the primary metabolic and regulatory events during conidiation, we sequenced the transcriptome of *Bgt* epiphytic structures at 3 (vegetative hyphae growth), 4 (foot cells initiation), and 5 (conidiophore erection) days post-inoculation (dpi). RNA-seq analyses identified 556 and 404 (combined 685) differentially expressed genes (DEGs) at 4 and 5 dpi compared with their expression levels at 3 dpi, respectively. We found that several genes involved in the conversion from a variety of sugars to glucose, glycolysis, the tricarboxylic acid cycle (TAC), the electron transport chain (ETC), and unsaturated fatty acid oxidation were activated during conidiation, suggesting that more energy supply is required during this process. Moreover, we found that glucose was converted into glycogen, which was accumulated in developing conidiophores, indicating that it could be the primary energy storage molecule in *Bgt* conidia. Clustering for the expression profiles of 91 regulatory genes showed that calcium (Ca^2+^), H_2_O_2_, and phosphoinositide (PIP) signaling were involved in *Bgt* conidiation. Furthermore, a strong accumulation of H_2_O_2_ in developing conidiophores was detected. Application of EGTA, a Ca^2+^ chelator, and trifluoperazine dihydrochloride (TFP), a calmodulin (CaM) antagonist, markedly suppressed the generation of H_2_O_2_, affected foot cell and conidiophore development and reduced conidia production significantly. These results suggest that Ca^2+^ and H_2_O_2_ signaling play important roles in conidiogenesis and a crosslink between them is present. In addition to some conidiation-related orthologs known in other fungi, such as the velvet complex components, we identified several other novel *B. graminis*-specific genes that have not been previously found to be implicated in fungal conidiation, reflecting a unique molecular mechanism underlying asexual development of cereal powdery mildews.

## Introduction

*Bgt* and *Blumeria graminis hordei* (*Bgh*) are economically important pathogens of cereals that can cause devastating damage to wheat and barley (Dean et al., 2012). Diseased plants display a white powdery mold on the leaves and stems and suffer from deprivation of nutrients by these fungi. *B. graminis* can reproduce not only by asexual conidia, but also by sexual crossing, which take places between two isolates with opposite mating types to generate ascospores. These two types of spores serve as primary inoculums and are spread by wind. Fungicides and resistant cultivars can be utilized in the management of these diseases. Both *Bgt* and *Bgh* are obligate biotrophic parasites that can complete their life cycles only on living hosts, and this characteristic limits the advancement of molecular functional analyses by genetic transformation. Nevertheless, significant progress in these analyses has been accomplished via global large-scale (“-omics") approaches such as genomics, transcriptomics, proteomics, and metabolomics (Bindschedler et al., 2016). Insights from these studies provide useful information for future genetic resistance improvement and fungicide development used in protection from these diseases.

Conidiation is one of the most important modes of reproduction in powdery mildews (Glawe, 2008). This process usually occurs several times during a growing season of plants, yet sexual recombination often takes place only once a year. Haplogroup analysis of the *Bgt* genome revealed that clonal reproduction occurs more frequently than sexual recombination (Wicker et al., 2013). Large numbers of the conidia of *Bgh* can be produced within several hours (Jørgensen and Torp, 1978), and can be transmitted by wind from continental Europe to Britain (Brown and Hovmoller, 2002), contributing to population migration and rapid spread of mildew disease. Therefore, understanding their molecular mechanism of conidiogenesis of *B. graminis* may be helpful in developing better strategies for control of these diseases.

The conidiogenesis process of *B. graminis* consists of the formation of foot cells, the development of conidiophores and then the release of conidia. Similar to other fungi, like *Aspergillus nidulans* and *Magnaporthe oryzae*, cereal powdery mildews must go through a period of vegetative hyphae growth after successful infection. Subsequently, conidiation typically begins when foot cells arise from vegetative hyphae and then conidiophores are erected by repeated elongation and septation of foot cells to produce massive conidia (Moriura et al., 2006). Moreover, new conidia successively form on the previous conidia generated from foot cells, thereby forming conidia chains (Glawe, 2008). Thus, foot cells control the shift from vegetative growth to asexual development and the speed of new conidial cell production of *B. graminis*. Uncovering the changes in metabolism and regulation during foot cell and conidiophore differentiation will contribute to better understanding the conidiation mechanism of powdery mildews.

Despite the agricultural importance of *B. graminis*, little work has been done to explore the genetic basis of asexual sporulation in powdery mildews. *Bgh* undergoes a series of dynamic changes in carbohydrate and lipid metabolism during asexual reproduction (Both et al., 2005). cDNA amplified fragment length polymorphism analysis identified 620 differentially expressed fragments related to metabolism and signaling during conidiation of *Erysiphe necator* (Wakefield et al., 2011). Conversely, significant advances in studies on asexual reproduction with an emphasis on regulatory factors have been made in several other fungi, indicating that there is a central regulatory pathway, the BrlA pathway, involved in conidiogenesis regulation of *A. nidulans* and *A. fumigatus*. Moreover, some upstream development activators (e.g., FluG, FlbC, FlbD, and FlbE) and downstream development regulators (e.g., velvet complex consisting of VosA, VelB, VelC, and LaeA) also act in regulating conidiation (Park and Yu, 2012; Alkhayyat et al., 2015). Additionally, at least two G protein mediated signal transduction pathways are involved in *A. nidulans* conidiation by negatively regulating the BrlA pathway (Yu, 2006). Conidiation-related heterotrimeric G protein signaling components include G protein coupled receptors (GPCRs), heterotrimeric G proteins and regulators of G protein signaling proteins (Park and Yu, 2012; Wang et al., 2013). Recently, a few small monomeric GTPases have also been shown to be implicated in various cellular processes including conidiation and tolerance to multi-stressors such as H_2_O_2_ (Guan et al., 2015). External signals from upstream G protein signaling can be transduced to some downstream central regulatory pathways that govern conidiation. It is known that the Ca^2+^ mediated signaling pathway (Nguyen Q.B. et al., 2008; Li et al., 2015), mitogen-activated protein kinase (MAPK) signaling (Chen et al., 2016) and reactive oxygen species (ROS) signaling (Chung, 2012; Viefhues et al., 2014) are also correlated with conidiogenesis. Furthermore, the crosslink between MAPKs cascade and Ca^2+^ signaling as well as that between MAPKs cascade and ROS signaling have been found in conidiation regulatory pathways (Huang et al., 2015; Wei et al., 2016).

The main purpose of this study was to identify the central metabolic and regulatory events during sporulation of *Bgt*. The characteristics of obligate biotrophy that underpin *B. graminis* development have historically made it difficult to design experiments that expand our understanding of these processes. Here, we performed a RNA-seq analysis of the epiphytic structures of *Bgt* during conidiation and conducted additional histological and pharmacological investigations. The resulting data provide new insights into the molecular mechanism underpinning conidiogenesis of *B. graminis*.

## Materials and Methods

### *Bgt* Isolate, Wheat Line, and Inoculation

*Bgt* isolate 21-2, collected in Jiangsu province of China in 2011, and Chancellor, a susceptible line without powdery mildew (*Pm*) resistance gene (Briggle, 1969) were used in this study. Plant growth and pathogen inoculation were conducted according to the method described previously (Zeng et al., 2014).

### Sample Collection and RNA Preparation

The epiphytic structures of isolate 21-2, growing on leaf segments of Chancellor at 3, 4, 5 dpi were embedded in the acetate cellulose and dissected with a tweezer. These samples were chilled in liquid nitrogen immediately and stored at -80°C. Total RNA of samples with three biological replicates at each time point was extracted using the RNAiso Plus Purification Kit (TAKARA, Japan) following the manufacturer’s instruction. After RNA purity was checked using the NanoDrop ND-1000 spectrophotometer (Thermo Scientific, Wilmington, DE, United States), the Qubit^®^ RNA Assay Kit in Qubit^®^2.0 Flurometer (Life Technologies, Foster City, CA, United States) was employed to determine concentration of these RNA samples. Then RNA integrity was confirmed by the Agilent 2100 Bioanalyzer (Agilent Technologies, Santa Clara, CA, United States). Two sets of qualified RNA samples were obtained under the same conditions. One set was used for RNA sequencing, while the other set was used for quantitative reverse-transcriptase PCR (qRT-PCR).

### Libraries Construction and Sequencing

Before mRNA sequencing, poly (A) mRNA was isolated from total RNA using the NEB-E7490 Poly (A) mRNA Magnetic Isolation Kit (NEB, United States), and then fragmented by the Fragmentation Kit (Ambion, Austin, TX, United States). cDNA libraries were constructed using the NEBNext^®^Ultra^TM^ RNA Library Prep Kit for Illumina^®^(NEB, United States) according to the manufacturer’s recommendations. Briefly, the first-strand and the second of cDNA were synthesized using M-MuLV reverse transcriptase and DNA polymerase I (Invitrogen), respectively. After NEBNext adaptors were ligated to the adenylated 3′ ends of cDNA, fragments of approximately 500 bp in length were purified and selected with the AMPure XP system (Beckman Coulter, Beverly, MA, United States). Next, these fragments were enriched by PCR amplifications and library quality was assessed by the Agilent Bioanalyzer 2100 System. RNA sequencing was performed using a paired-end 100 (each end with 100 bases) strategy on the Illumina Hiseq 2500 platform at Biomarker Technology, Co., Ltd (Beijing, China) to generate 2 billion bases per sample. We deposited the raw data in the NCBI sequence read archive under accession number SRP092295.

### Reference Genome-Based Reads Mapping and Quantitative Analysis of Gene Expression

All reads of nine libraries were mapped to the reference genome of *Bgt* isolate 96224 (Wicker et al., 2013) using the Tophat2 software (Kim et al., 2013), allowing up to two base mismatches. To quantify the transcript abundance of each expressed gene, fragments per kilobase per million reads (FPKM) values were calculated by the Cufflinks software (Trapnell et al., 2010). Transcript abundances among different libraries were compared using the DESeq software (Anders and Huber, 2010) and the multiple hypothesis test was conducted to check the difference significance of gene expression with a *p*-value. The *p*-values were adjusted using the Benjamini and Hochberg’s approach by controlling the false discovery rate (FDR) < 0.01. Genes with an adjusted *p*-value < 0.05 and | expression fold change|≥ 2 were deemed with defined DEGs. In addition, the Pearson correlation coefficients (*r*^2^) of FPKM between replicates were calculated using the method of Simple Error Ratio Estimate (Schulze et al., 2012).

### DEGs Annotation

To characterize the detailed functions of DEGs, nucleotide sequences were aligned against various protein databases including the SwissProt database and the NCBI non-redundant (Nr) database via BLASTX (threshold set to E-value < 1e-5). The BLASTX results were analyzed using the Blast2GO^1^ software (Gotz et al., 2008) to conduct a Gene Ontology (GO) functional analysis. Annotations were enriched and refined using the TopGo package^2^. The DEGs were also mapped to the Clusters of Orthologous Groups (COG) database (Tatusov et al., 1997) to predict and classify functions, and to the KEGG database^3^ to assign metabolic pathways. For enrichment analysis of GO terms and Kyoto Encyclopedia of Genes and Genomes (KEGG) pathways, two tailed *p*-values were obtained by Fisher’s exact test. Terms and pathways with FDR corrected *p*-values < 0.05 were considered to be significantly enriched.

### qRT-PCR Validation

To confirm the reliability of transcript levels that were quantified based on the ratio of FPKM values, the relative expression levels of 22 DEGs were checked using qRT-PCR evaluation. After having been digested by DNase I (RNase free, TAKARA, Japan), first-strand cDNAs were synthesized from 1 μg total RNA using the PrimeScript^TM^ First-strand cDNA Synthesis Kit (TAKARA, Japan) following the manufacturer’s protocol. Specific primers for each gene used in qRT-PCR were listed in Supplementary Table S1. All qRT-PCR reactions were conducted using the SYBR Green I Nucleic Kit (Life Technologies, United States) and the ABI 7500 real-time PCR system (Applied Biosystems, United States) with the following programs: 95°C for 10 min, followed by 40 cycles of 95°C for 10 s and 60°C for 30 s. The house-keeping gene encoding beta-tubulin was used as the internal control (Hacquard et al., 2013). All PCR amplifications were run in triplicates. The 2^-ΔΔCT^ method (Livak and Schmittgen, 2001) was used to calculate the relative expression levels.

### Identification of Genes Involved in Primary Metabolic Processes and Regulatory Pathways and *B. graminis*-Specific Proteins

To identify genes acting in primary metabolism among DEGs, we extracted genes that are affiliated with annotations in the COG database corresponding to carbohydrate transport and metabolism (E), energy production and conversion (C) and lipid transport and metabolism (I) at first. Additional genes were identified based on associated functional annotations in the GO, the KEGG, SwissProt, and Nr databases. All these genes were combined and checked by BLASTP search in the GenBank manually.

To identify putative signaling proteins, we analyzed major fungal signaling pathways including heterotrimeric G proteins/GPCRs, small G proteins, Ca^2+^, PIP, and H_2_O_2_ mediated signaling. Since almost 92% of predicted *Bgt* genes have homologs in *Bgh* genome (Wicker et al., 2013), protein sequence similarity alignments against putative signaling proteins in *Bgh* genome (Kusch et al., 2014) were performed by BLASTP. All hits with a cutoff of E-value < 1e-10 were considered significant. Subsequently, additional candidates (such as members involved in PIP and H_2_O_2_ mediated signaling) were identified by searching associated annotations in the GO, KEGG, SwissProt, Nr, and InterPro databases. GPCR candidates were further corroborated by the presence of six to eight trans-membrane domains^4^. For transcription factor (TF) mining, query sequences for BLASTP were obtained from the Fungal Transcription Factor Database^5^ (Park et al., 2008). Additional TFs were also predicted based on their annotations in the database as described above. Finally, a manual inspection using BLASTP search in the NCBI website was conducted to check the combined results gene by gene.

To identify the proteins specific to *Bgt* or *Bgh*, we conducted BLASTP searches against the Nr database using standard parameters. The proteins without significant hits (E-value ≥ 1e-5 to proteins in other fungi, except *Bgh*) were considered as *B. graminis*-specific proteins. To obtain more annotations for these *B. graminis*-specific proteins, BLASTP alignments against the proteins of *Bgh* isolate DH14 (Spanu et al., 2010) were performed (E-value cutoff 1e-10).

### EGTA and TFP Treatment

EGTA (GEN-VIEW company, United States), was dissolved in distilled water (pH8.0). TFP (TCI company, Shanghai, China) was added to distilled water to make a 100 mM stock solution. The surfactant Tween 20 was added to make the final concentration 0.025%. The same volume of 0.025% Tween 20 was used as a negative control. Detached leaf segments were inoculated and incubated under conditions described above. A 2.4 mL solution was sprayed on the infected leaf segments using the Potter spraying tower at pressure of 0.25 MPa. After air-drying for 1 h at 25°C, incubation of the treated leaf segments resumed under the condition used before treatment.

### Staining and Histological Observation

Leaf segments of Chancellor infected by isolate 21-2 were stained with alcoholic lactophenol trypan blue (Yang et al., 2008). The Lugol reagent (KI and I_2_) staining method (Both et al., 2005) was employed to detect glycogen generation. After decoloration in 95% ethyl alcohol, inoculated leaf segments were stained with the Lugol reagent for 10 min at room temperature and observed immediately. To observe the generation of H_2_O_2_ in this pathogen, *Bgt*-infected leaf segments were stained with 3, 3′- diaminobenzidine (DAB, Sigma-Aldrich, St. Louis, MO, United States) (Thordal-Christensen et al., 1997). Leaf segments were briefly soaked in 1mg/ml DAB solution (pH3.8), at 25°C for 8 h and decolored with clearing solution (ethanol: acetic acid: ddH_2_O = 94:4:2, v/v) for 1 h.

To count the number of foot cells at 5 dpi and conidiophores at 6 dpi in single colonies, 30 colonies on each of three leaf segments were randomly selected and observed through a light microscope at 400× magnification. Mycelium expansion width of 30 colonies on each of three leaf segments at 5 dpi was measured using a micrometer at 100× magnification. To evaluate the impact of the treatments with EGTA or TFP on H_2_O_2_ production, the percentage of colonies with stained conidiophore (s) at 6 dpi were investigated by scoring the number in 30 randomly chosen colonies on each of three leaf segments. To measure conidia production, each of three 2.5 cm long leaf segments collected at 10 dpi was put into 2.5 mL 0.025% Tween 20 to produce a suspension of spores. Concentration of spores was estimated using a hemocytometer at 100× magnification (Gadoury et al., 2012). These experiments were conducted two times independently.

### Statistical Analysis

The analysis of variance was performed to determine whether the error variances of treatment and untreated control were homogeneous. The significance of the differences was evaluated using Student’s *t*-tests.

## Results

### Asexual Development of *Bgt*

**FIGURE 1:**
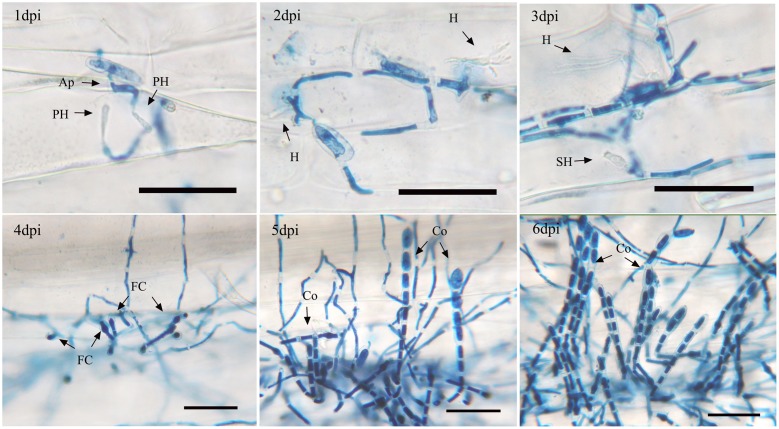
Asexual life cycle of *Bgt*. Infected leaf segments of Chancellor by isolate 21-2 were sampled at 1, 2, 3, 4, 5, and 6 day post inoculation (dpi) and trypan blue staining was performed. Ap, appressorium; PH, primary haustorium; H, the first mature haustorium; SH, secondary haustorium; M, mycelium; FC, foot cell; Co, conidiophore. The bars represent 50 μm.

The asexual life cycle of isolate 21-2 on Chancellor was observed under a light microscope (**Figure 1**). At the early infection stage, conidia with appressorium generated primary haustoria underneath the infected sites by 1 dpi and haustoria matured by 2 dpi. Then secondary haustoria began to form in the epidermis and vegetative hyphae proliferated on leaf surface by 3 dpi. Subsequently, some swollen cells, called foot cells, appeared on vegetative hyphae by 4 dpi and many foot cells could be observed by 5 dpi. The upper portion of foot cells then began to elongate and repeated septation separated a few conidial cells, which compose conidophores, the specialized developmental structures for conidia generation. By repeating this process, several conidophores started to erect on foot cells in single colonies by 5 dpi and the upper conidial cells enlarged during maturation. By 6 dpi, clustered conidiophores were seen in single colonies. Finally, mature spores were gradually released from the conidiophores.

### High-Quality RNA-seq Data Obtained from Epiphytic Tissues of *Bgt*

To analyze changes in the transcription profiles of *Bgt* in the transition from vegetative growth to asexual reproduction, the epiphytic materials at 3, 4, and 5 dpi were sampled for transcriptome sequencing. Three biological replicate samples were collected for each phase. For mRNA sequencing, nine cDNA libraries were constructed and then a total of 23.05 Gb of clean data composed of 91,495,902 pair-end reads was generated. Q30% of these clean data from each library were more than 87.8% (**Table 1**), indicating that these Illumina reads were of a high quality. Reads mapping showed that a range from 73.81 to 86.66% reads of these libraries were successfully mapped to the reference genome of *Bgt* isolate 96224 (Wicker et al., 2013) (Supplementary Table S2). Among the matched reads more than 65% of the reads from the combined data were mapped to exonic regions and almost all of the rest were mapped to intergenic regions (Supplementary Figure S1). Saturation analysis indicated these libraries were sequenced to saturation and represented the transcripts in the conditions tested (approximate 1G clean data, Supplementary Figure S2). Correlation clustering analysis based on FPKM values suggested that our RNA-seq data were highly reproducible (Supplementary Figure S3).

**Table 1:** Descriptions of nine RNA-seq libraries of *Bgt* during development on Chancellor at 3, 4, and 5 dpi.

Development stage name	Library name	Total reads	Total bases	GC content	Q20(%)	Q30(%)
C3 dpi	C3 dpi-1	9,840,112	2,478,911,943	45.18%	98.26	93.6
	C3 dpi-2	8,426,123	2,122,690,344	45.06%	98.39	94.1
	C3 dpi-3	9,978,332	2,513,839,296	45.15%	98.23	93.5
C4 dpi	C4 dpi-1	9,528,424	2,400,489,205	44.94%	98.29	93.72
	C4 dpi-2	9,737,953	2,453,181,685	44.90%	98.26	93.55
	C4 dpi-3	9,028,747	2,274,575,346	45.04%	98.38	94.13
C5 dpi	C5 dpi-1	12,397,492	3,123,152,835	45.06%	93.4	88.26
	C5 dpi-2	12,580,842	3,169,216,532	45.01%	93.39	88.24
	C5 dpi-3	9,977,877	2,513,780,503	45.30%	93.19	87.89

*Development stage name: a time course of *Bgt* infection to Chancellor. Library name: RNA samples of the epiphytic structures growing on leaf segments of Chancellor at 3, 4, 5 dpi with three biological replicates for library construction; Total reads: number of total pair-end reads in each clean data; Total bases: total base number in each clean data; GC content: percentage of G and C in clean data; Q20(%): percentage of bases with quality value more than 20 in each clean data; Q30(%): percentage of bases with quality value more than 30 in each clean data.*

To further confirm the reliability of our RNA-seq expression data measured by FPKM fold changes, we selected 22 DEGs and monitored their expression levels using qRT-PCR. These proteins included nine functional proteins, 10 regulatory factors, and three hypothetical proteins with unknown functions. The relative transcript levels determined by qRT-PCR and RNA-seq were positively correlated with a Pearson coefficient *R*^2^ = 0.777 (Supplementary Figure S4). These results support the validity of our RNA-seq data and can be used for downstream analysis.

### Differential Expression Analysis of Genes Regulated during Conidiation

**FIGURE 2:**
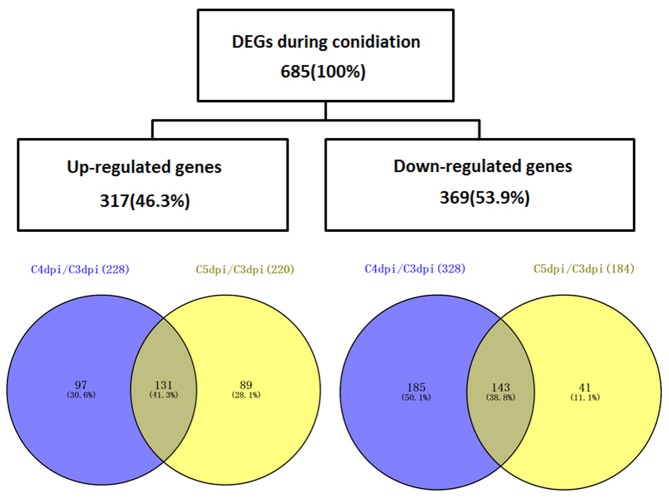
A *Venn* diagram illustrating the numbers of DEGs of *Bgt* during conidiation on Chancellor. The overlaps of DEG sets obtained from the comparison C4 dpi/C3 dpi and C5 dpi/C3 dpi are shown.

To analyze the changes in gene expression across different developmental stages, we performed differential expression analysis at three different time points (3, 4, and 5 dpi) using the DESeq software (**Table 2**). 556 and 404 genes were differentially expressed in the comparisons of C4 dpi/C3 dpi, and C5 dpi/C3 dpi, respectively (Supplementary Table S3). Thus we found a total of 685 DEGs, of which 317 genes were up-regulated and 369 were down-regulated (**Figure 2**). Within these genes, 275 (including one gene coding a protein, EPQ64082.1, down-regulated at 4 dpi but up-regulated at 5 dpi) were differentially expressed in both comparison pairs. Moreover, 281 and 129 specific in the library pairs of C4 dpi/C3 dpi and C5 dpi/C3 dpi, were related to the foot cell and conidiophore development, respectively.

**Table 2:** Differentially expressed genes (DEGs) and annotated gene number during conidiation of *Bgt* on Chancellor.

Pairwise comparison	DEG number	Up-regulated	Down-regulated	KEGG	GO	COG	SwissProt	Nr
C4 dpi/C3 dpi	556	228	328	134	127	181	340	556
C5 dpi/C4 dpi	131	115	16	22	27	35	75	131
C5 dpi/C3 dpi	404	220	184	90	80	128	235	404

*To obtain annotations for DEGs of three library pairs, we mapped them to the KEGG, GO, COG, SwissProt, and NCBI Nr databases.*

To reveal the potential functions of the 685 DEGs, the COG, GO, and KEGG databases were used to analyze functional classification and pathways. Among the 25 COG categories, except for the cluster for “general function prediction only,” the largest group was the cluster for “carbohydrate transport and metabolism” in both pair-comparisons (26 genes in C4 dpi/C3 dpi and 21 genes in C5 dpi/C3 dpi), followed by the clusters for “post-translational modification, protein turnover, chaperones,” “lipid transport and metabolism,” “amino acid transport and metabolism,” and “energy production and conversion” (Supplementary Figure S5).

GO enrichment analysis was performed with the 685 DEGs (Supplementary Table S4). The analysis identified three significantly enriched GO terms, with FDR corrected *p*-value < 0.05, including the GO categories of “oxidoreductase activity” (GO:0016491), “glycogen biosynthetic process” (GO:0005978) and “hexose catabolic process” (GO:0019320) (Supplementary Figure S6). The DEGs involved in glycogen biosynthesis and hexose catabolism were specifically enriched in the comparison of C5 dpi/C3 dpi. To gain more information, we also inspected GO terms with non-corrected *p*-value < 0.05 (Fisher’s exact test). Thus additional GO categories such as “carbohydrate metabolic process” (GO:0005975), “cell redox homeostasis related process” (GO:0045454; GO:0034599), and “oxidation–reduction process” (GO:0016491) were found in both comparisons of C4 dpi/C3 dpi and C5 dpi/C3 dpi (Supplementary Figure S6). Collectively, DEGs corresponding to these GO terms are mainly involved in carbohydrate metabolism and cell redox process.

Using KEGG pathway enrichment analysis, we categorized the biological functions of the 685 DEGs. No significant enriched pathway with FDR corrected *p*-value < 0.05 was observed between library pairs (Supplementary Table S5). As with the GO analysis, pathways with non-corrected *p*-value < 0.05 (Fisher’s exact test) were also taken in account. This analysis showed that the pathway of “peroxisome” (ko04146) was highly represented in both pair-comparisons. Six DEGs were observed in the “peroxisome” pathway, which can be classified into three functional groups, peroxisome biogenesis (two peroxisomal proteins, EPQ61877.1 and EPQ67652.1), fatty acid oxidation (two enzymes, EPQ67195.1 and EPQ66210.1), and antioxidant system (two scavengers, EPQ63972.1 and EPQ62743.1) (Supplementary Figure S7). The results indicate that fatty acid and ROS metabolism might be involved in conidiation.

### DEGs of *Bgt* Involved in Carbohydrate Metabolism, Energy Production, and Unsaturated Fatty Acid Metabolism during Conidiation

**FIGURE 3:**
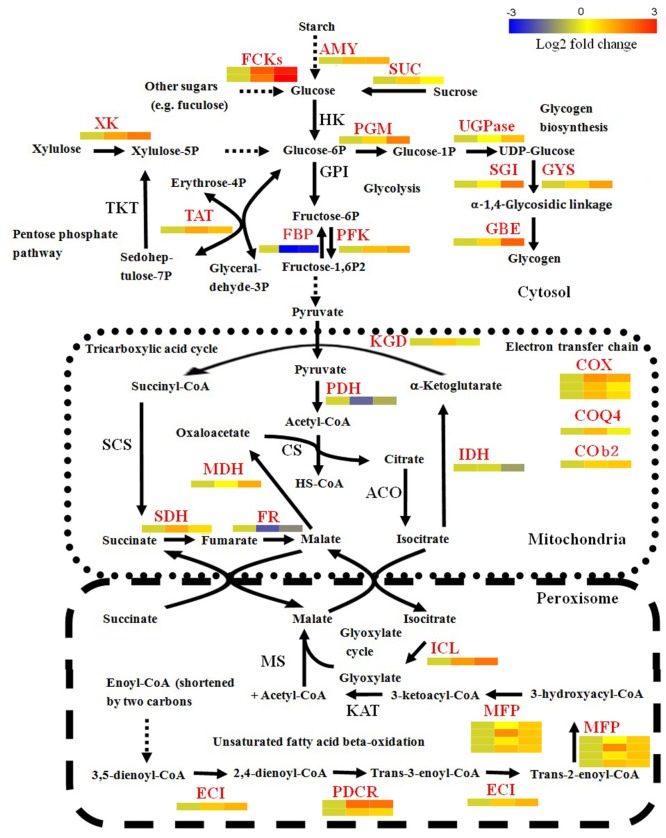
Transcriptional changes of genes involved in basic metabolism in *Bgt* during asexual development on Chancellor. DEGs are shown in red font. The relative expression levels at 3, 4, and 5 dpi are indicated using heat maps below or aside these genes. Solid and dotted arrows indicate one-step and multiple-step reactions during the catalytic process, respectively. The detail information of these genes is listed in Supplementary Table S3. AMY, α-amylase; FCK, FGGY carbohydrate kinase; SUC, sucrase; HK, hexokinase; GPI, glucose-6-phosphate isomerase; PFK, phospho-fructokinase; FBP, fructose-1,6-bisphosphatase; PGM, phosphoglucomutase; UGPase, UDP-glucose pyrophosphorylase; GYS, glycogen synthase; GBE, glycogen branching enzyme; SGI, self-glucosylating initiator of glycogen synthesis; XK, xylulose kinase; TKT, transketolase; TAT, transaldolase; KGD, α-ketoglutarate dehydrogenase; PDH, pyruvate dehydrogenase complex; IDH, isocitrate dehydrogenase; ACO, aconitase; MDH, malate dehydrogenase; CS, citrate synthase; SDH, succinate dehydrogenase; FR, fumarase; COX, cytochrome c oxidase complex; COQ4, ubiquinone biosynthesis protein COQ4; COb2, cytochrome b2 L-lactate cytochrome-c oxidoreductase; ICL, isocitratelyase; MS, malate synthase; KAT, β-ketoacyl-CoA thiolase; MFP, multifunctional enzyme of the peroxisomal fatty acid beta-oxidation pathway (carrying putative function of enoyl-CoA hydratase, 3-hydroxyacyl-CoA dehydrogenase and 3-hydroxyacyl-CoA epimerase); ECI, enoyl CoA isomerase; PDCR, peroxisomal 2,4-dienoyl-CoA reductase.

**FIGURE 4:**
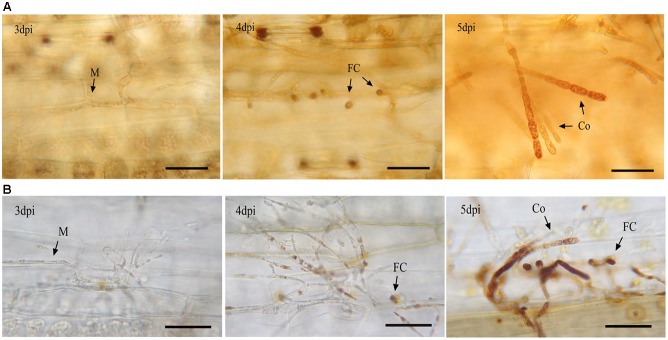
Lugol staining of glycogen **(A)** and DAB **(B)** staining of H_2_O_2_ in *Bgt* during asexual reproduction. Infected leaf segments were sampled at 3, 4, and 5 dpi. H_2_O_2_ and glycogen can be identified as brown complex. M, mycelium; FC, foot cell; Co, conidiophore. The bars represent 50 μm. H_2_O_2_ and glycogen began to appear in the foot cells by 4 dpi and largely accumulated in the developing conidiophores by 5 dpi.

Since many DEGs were found to be involved in basal metabolic processes, we used BLAST to search the 685 DEGs against the SwissProt and NCBI Nr databases to further identify enzymes involved in the carbohydrate, energy, and lipid metabolism. The results are listed in Supplementary Table S6. This analysis identified 18 carbohydrate metabolism-related genes. Within these genes, eight genes play roles in metabolizing various sugars into glucose or glucose phosphate. Most of them (7/8) were significantly up-regulated at 4 and/or 5 dpi (**Figure 3**). For example, two FGGY carbohydrate kinases, which can catalyze phosphorylation of several sugars and their products, connected to glycolysis and the pentose phosphate pathway (Zhang et al., 2011), displayed more than eightfold increases of mRNA levels in the library of C5 dpi. The substrates catalyzed by the seven enzymes included β-1,3-glucan, starch, sucrose, xylulose and fuculose, suggesting that conversion of diverse sugars into glucose become active during conidiation. Furthermore, the expression profiles of genes associated with glycolysis (EPQ67062.1, up-regulated), the pentose phosphate pathway (EPQ66083.1, up-regulated), and gluconeogenesis (EPQ64945.1, down-regulated), suggest that generated glucose might partially be utilized as fuel during *Bgt* conidiation (**Figure 3**). In addition, we found five genes related to glycogen biosynthesis to be up-regulated at 5 dpi (**Figure 3**). This indicates that glycogen synthesis may be activated in conidiophores. To further confirm this result, we performed an iodine staining assay to test whether glycogen was accumulated in conidiophores. As predicted, glycogen began to generate in the foot cells at 4 dpi and accumulated at high levels in the developing conidiophores at 5 dpi (**Figure 4A**).

For energy metabolism, 14 genes related to energy production and transport showed differential expression during conidiation. Among these genes, 11 genes, which are mainly involved in the TAC, the glycoxylate cycle, energy transport and the ETC (**Figure 3**), are significantly up-regulated. These data suggest that energy metabolism is enhanced during conidiation of *Bgt*.

For lipid metabolism, a total of 29 DEGs were obtained in our transcriptomic analysis. Most DEGs (8/11) involved in triacylglycerol and phosphatidate degradation were significantly down-regulated during sporulation. However, seven genes related to the break down pathway of peroxisomal unsaturated fatty acid, such as peroxisomal 2,4-dienoyl-CoA reductase, enoyl-CoA isomerase (Wanders and Waterham, 2006) and the multifunctional enzymes of the peroxisomal fatty acid beta-oxidation pathway (Nguyen S.D. et al., 2008) were up-regulated (**Figure 3**). Moreover, two genes acting in the glyoxylate cycle and two in the carnitine transport, where acetyl CoA is transported from peroxisome to mitochondria (van Rossum et al., 2016), were also activated (**Figure 3**). These results suggest that lipid degradation is repressed, while unsaturated fatty acid oxidation is enhanced. We did not find evidence of the activation of enzymes responsible for lipid synthesis. However, a significant increase of the expression level of one gene coding the caleosin protein (EPQ66680.1), which is related to storage lipid bodies, was detected.

### Activation of Antioxidant Redox System and Accumulation of H_2_O_2_ in Conidiophores of *Bgt*

Based on the results from the GO and KEGG analysis, we hypothesized that cell redox homeostasis-related processes might be correlated to *Bgt* conidiation. Therefore, we looked for DEGs with annotations associated with ROS clearance and production in the SwissProt, Nr, and InterPro databases. This analysis resulted in 14 genes coding for putative antioxidant proteins, including two superoxide dismutases, one catalase, two peroxiredoxins, five thioredoxins, two glutathione oxidoreductases, and two glutaredoxins. All these genes, except for the catalase coding gene, exhibited up-regulation patterns at 4 or/and 5 dpi, which indicates that the antioxidant system for some ROS, such as H_2_O_2_, is activated during sporulation. For ROS-producing enzymes, only one putative NADPH oxidase A (NoxA, EPQ61671.1), tightly linked with H_2_O_2_-generating reactions in conjunction with superoxide dismutases, was significantly down-regulated at 4 dpi.

To assess if H_2_O_2_ accumulated in sporulating tissues, a DAB staining experiment was carried out. As shown in **Figure 4B**, H_2_O_2_ began to appear in mycelium and foot cells at 4 dpi and bursted in the developing conidiophores at 5 dpi. These results indicate that H_2_O_2_ metabolism is correlated with the conidiophore development. By contrast, low quantities of H_2_O_2_ were detected in the infected epidermal cells during the time-course of 3, 4 and 5 dpi, suggesting that little H_2_O_2_ was generated by the host cells.

### DEGs Involved in Major Regulatory Pathways during *Bgt* Conidiation

**FIGURE 5:**
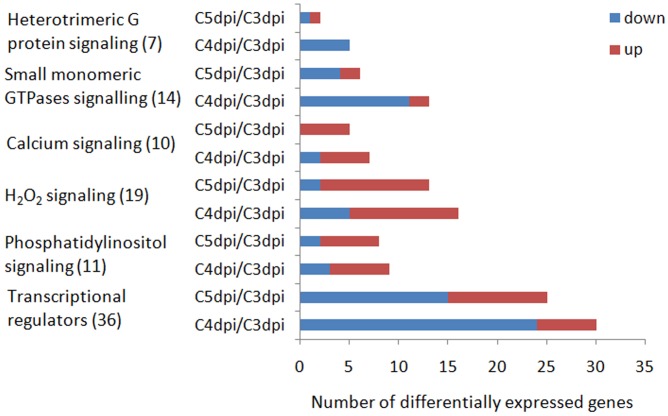
Number of DEGs coding for putative signaling proteins and transcriptional regulators up-regulated or down-regulated during conidiation of *Bgt*. Numbers in the brackets are the corresponding number of genes in each signaling pathway or function category. Up and down represent up-regulation and down-regulation, respectively.

**FIGURE 6:**
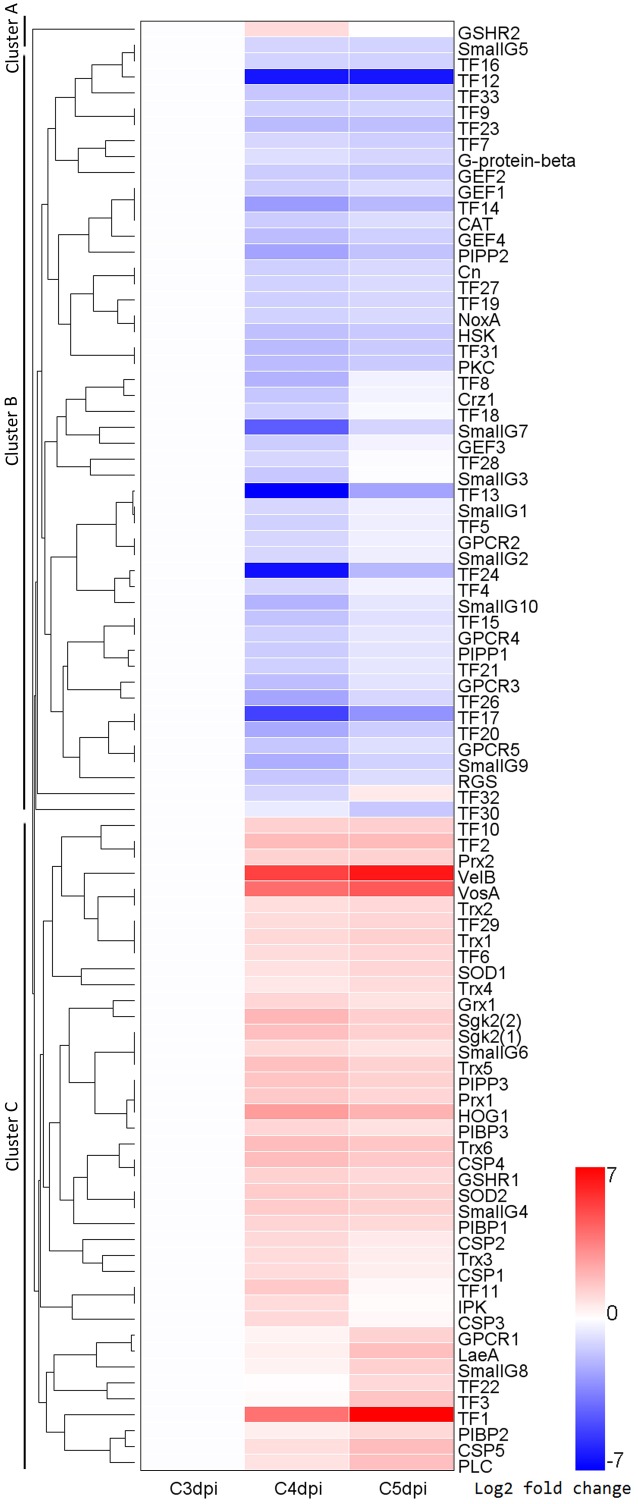
A heatmap for the expression patterns of 91 putative regulator coding genes in *Bgt* at 3, 4, and 5 dpi. Regulatory pathways in which these regulators participated in include the G protein signaling, small G protein signaling, calcium signaling, H_2_O_2_ signaling, PIP signaling and transcriptional regulation. A hierarchical clustering analysis revealed three major clusters, Cluster A, Cluster B, and Cluster C. The detail information of these genes is listed in Supplementary Table S7. G-protein-beta, heterotrimeric G protein beta subunit; RGS, regulator of G protein signaling; GPCR, G protein coupled receptor; SmallG, small G protein; GEF, guanine nucleotide exchange factor; SOD, superoxide dismutase; CAT, catalase; Prx, peroxiredoxin; Trx1, thioredoxin; GSHR,*glutathione oxidoreductase; Grx, glutaredoxin; NoxA, NADPH oxidase A; HSK, histidine kinase; HOG1, protein kinase in high osmolarity glycerol pathway; CSP, calmodulin signal protein; Cn, calcineurin; Crz1, calcineurin-responsive zinc finger transcription factor; PIPP, phosphoinositide 3-phosphatase; PIBP, phosphatidylinositol binding protein; IPK, inositol polyphosphate kinase; PLC, phospholipase C, PKC, protein kinase C; Sgk2, serum and glucocorticoid inducible kinase2; VelB, VosA, LaeA, components of velvet complex; TF, transcription factor.*

To identify DEGs involved in major signaling pathways and transcriptional regulation, we used a combined strategy of sequence alignments against known regulators in other fungi and searches of genes with corresponding functional annotations in several databases (the GO, KEGG, SwissProt, Nr, and InterPro databases). The known regulators included those in *Bgh* (Kusch et al., 2014) and TFs in the Fungal Transcription Factor Database. In addition, prediction of transmembrane helices was performed in putative GPCRs. A total of 91 DEGs were identified, which are involved in a variety of regulatory pathways (**Figure 5** and Supplementary Table S7). The gene expression profiles were grouped based on their FPKM using hierarchical clustering. This analysis revealed three major clusters exhibiting distinct expression patterns during conidiation (**Figure 6**). Cluster A contained only one up-regulated glutathione oxidoreductase coding gene. Cluster B consisted mostly of members in G-protein signaling and some TF genes, which were down-regulated at 4 or/and 5 dpi. In contrast, genes in cluster C were markedly induced and most of them code for regulators in Ca^2+^, H_2_O_2_, and PIP signaling.

For the G protein signaling pathways, a total of 21 regulators were identified *in silico*. We found one heterotrimeric G protein, five GPCRs, and one RGS in the heterotrimeric G protein signaling pathway. For another type of G proteins, Small G proteins, we found nine putative GTPase activating proteins, one GTP binding protein, and four guanine nucleotide exchange factors. Among these 21 DEGs, 17 were down-regulated during sporulation and most down-regulation (11/17) took place at 4 dpi.

We also observed significant transcriptional changes associated with components of Ca^2+^ and PIP mediated signaling pathways, which can sense signals from the G protein signaling pathways. In this analysis, we found up-regulation of one phospholipase C (EPQ61894.1) coding gene. Since phospholipase C hydrolyses phosphatidyl inositol 4,5-bisphosphate to form inositol 1,4,5-trisphosphate (IP3) and diacylglycerol, two secondary messengers that involved in Ca^2+^ influx from other organelles or extracelluar space to cytosol, we hypothesize the cytoplasmic Ca^2+^ level might be increased. This is supported by two findings as follows: first, five CaM Ca^2+^ binding proteins coding genes were up-regulated at 4 and/or 5 dpi, because these proteins are the major transducers of cytoplasmic Ca^2+^ in eukaryotic cells. Second, protein sequence alignments revealed that a serine/threonine protein kinase (EPQ63189.1) showed a significant similarity to the Cmk1p (CCU77137.1), a putative Ca^2+^/CaM-dependent protein kinase (CCaMK) in *Bgh* (E-value = 1e-24). This gene also exhibited significantly increased mRNA levels at 4 and 5 dpi. These results suggest the changes in Ca^2+^ concentration are implicated in *Bgt* sporulation. However, no DEGs encoding for Ca^2+^ channel proteins, Ca^2+^ exchangers, Ca^2+^-transporting ATPases or calcipressin were found. In PIP mediated signaling, the increased mRNA levels of genes coding the phospholipase C and three PIP binding proteins suggest that PIP metabolism is enhanced on the cytosolic side of cell membranes.

To further analyze the DEGs involved in the cell response to H_2_O_2_ and signal transduction, we searched for putative regulators related to H_2_O_2_ signaling. In addition to the NoxA (EPQ61671.1) and 14 antioxidant proteins mentioned above, this analysis identified several other orthologous genes with putative roles in ROS signaling in *Alternaria alternata* (Chung, 2012), including one histidine kinase (HSK, EPQ67055.1), two TFs homologous with the redox-sensitive YAP1 (KZV08838.1) in yeast and a MAPK (EPQ63189.1) homologous to the protein kinase in high osmolarity glycerol pathway (HOG1) in *Bgh* (CCU77378.1). In total we identified 19 DEGs involved in ROS signaling, of which 14 were up-regulated at 4 or/and 5 dpi.

The *in silico* prediction of TFs resulted in 33 putative TF genes. The most abundant type were Zn finger proteins (14 candidates), followed by homeobox TFs (three candidates). Differential expression analysis indicated that 72.7% (24 of 33) of the TFs genes showed down-regulated expression patterns at 4 or/and 5 dpi. Notably, we found some genes showing similarities to known conidiation-related genes in other fungi including *BrlA*, *AbaA*, *WetA*, *FluG*, *FlbC*, *FlbD*, *VosA*, *VelB*, *VelC*, *LaeA*, *Vib-1*, and *Fluffy* (Park and Yu, 2012). Of these orthologous genes, *VosA* (EPQ63747.1), *VelB* (EPQ65573.1), *LaeA* (EPQ63484.1) and *Fluffy* (EPQ63997.1) were up-regulated, while *FlbC* (EPQ63575.1) and *Vib-1* (EPQ66091.1) were down-regulated. For example, the mRNA level of *VelB* was strongly elevated at 4 dpi with 33.4-fold and 5 dpi with 72.9-fold. The results suggest that the velvet complex (VosA, VelB, and LaeA) may play an important role in regulating conidiogenesis of *B. graminis*. *BrlA* (EPQ63980.1), *AbaA* (EPQ63911.1), *WetA* (EPQ64026.1), *FluG* (EPQ63351.1), and *VelC* (EPQ67732.1) displayed constitutive expression and *FlbD* (EPQ62684.1) were not expressed during sporulation.

### Effects of Ca^2+^ Chelator and CaM Antagonist on Conidiation of *Bgt* and Production of H_2_O_2_

**FIGURE 7:**
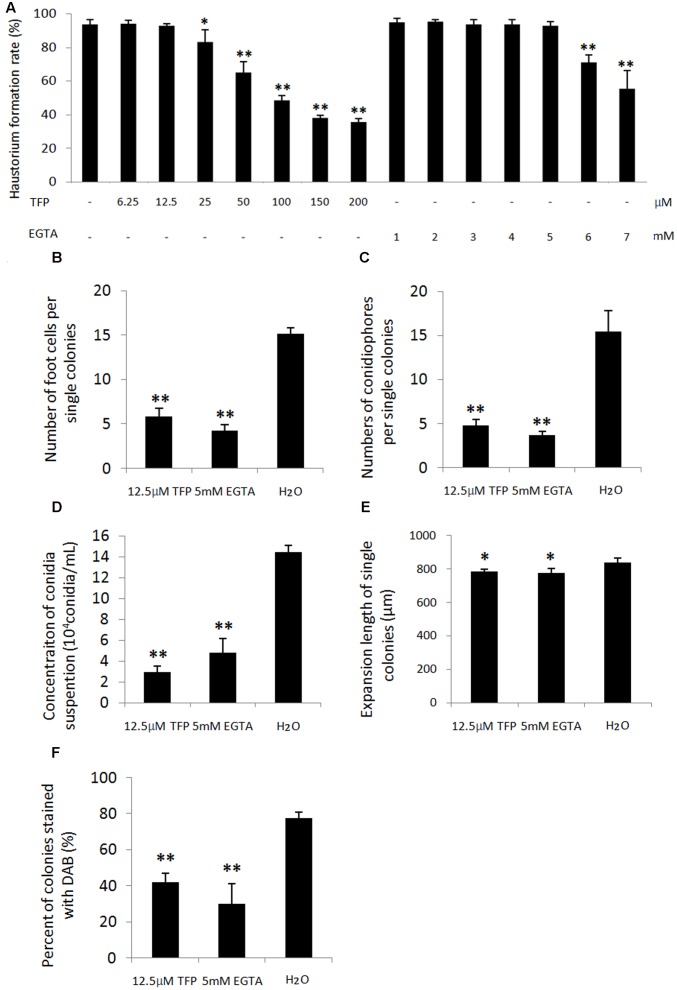
Effect of 5 mM EGTA and 12.5 μM trifluoperazine dihydrochloride (TFP) on conidiation and H_2_O_2_ accumulation during conidiation of *Bgt*. Wheat leaf segments of Chancellor were inoculated with isolate 21-2. A pilot experiment with a series concentration of two chemicals (dissolved in 0.025% Tween 20) was carried out by spraying at 1 dpi and the haustorium formation rates were investigated at 3 dpi **(A)**. Based on these data, 5 mM EGTA and 12.5 μM TFP were applied at 4 dpi by spraying the solutions. Infected leaf segments were sampled at 5 and 6 dpi and stained with the trypan blue and DAB solution. Next, a microscopy observation was carried out. Number of foot cells per single colonies at 5 dpi **(B)**, number of conidiophores per single colonies at 6 dpi **(C)**, conidia concentration of 2.5 mL conidiospore suspension dislodged from 2.5 cm long diseased leaf segments at 10 dpi **(D)**, mycelium expansion width of single colonies at 100× magnification at 5 dpi **(E)** and percentage of colonies with DAB stained conidiophore(s) at 5 dpi **(F)** were investigated with three replicates. For each replicate, 30 colonies were observed. All the experiments were performed two times with similar results. Data from another independent experiment are presented in Supplementary Figure S8. Values are means of data with three replicates of each treatment and error bars indicate standard error of the means. The significance of the differences between the treatments and untreated control was determined by Student’s *t*-tests. Significant decreases in the rates of foot cell and conidiophore formation and conidia production after 5 mM EGTA and 12.5 μM TFP treatment occurred (^∗∗^*p* < 0.01). Both treatments have a slight impact on the mycelium growth (^∗^*p* < 0.05).

**FIGURE 8:**
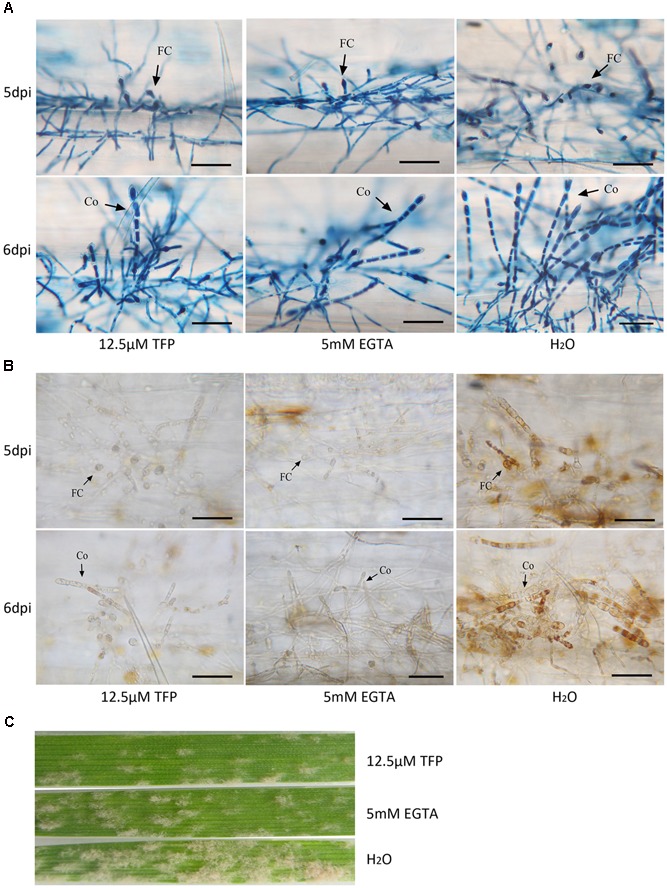
The foot cell and conidiophore development and H_2_O_2_ accumulation during *Bgt* sporulation on Chancellor when treated with 5 mM EGTA and 12.5 μM TFP. Microscopy pictures for the trypan blue **(A)** and DAB staining **(B)** are given. Diseased leaf segments were also photographed at 10 dpi **(C)**. Bar: 50 μm; FC, foot cell; Co, conidiophore.

To further analyze the role of Ca^2+^ and CaMs in regulating conidiation, EGTA, a extracellular Ca^2+^ chelator, and TFP, a CaM antagonist, were applied on wheat leaf infected with the powdery mildew isolate. A series of concentration of EGTA (1, 2, 3, 4, 5, 6, and 7 mM) and TFP (6.25, 12.5, 25, 50, 100, 200 μM) were sprayed at 1 dpi and the haustorium formation rates were investigated at 3 dpi. We found no significant difference of the haustorium formation rates between the treatments (1, 2, 3, 4, 5 mM EGTA; 6.25, 12.5 μM TFP) and untreated control (**Figure 7A**). However, when applied with 5 mM EGTA or 12.5 μM TFP at 4 dpi, the two inhibitors hampered foot cell and conidiophore formation significantly (*p* < 0.01, **Figures 7B,C**, 8A), thereby reduced conidia production significantly (*p* < 0.01, **Figures 7D**, **8C**). In addition, only a slight decrease of mycelium growth was observed (*p* < 0.05, **Figure 7E**). These results indicate that extracellular Ca^2+^ and intracellular CaMs are involved in *Bgt* conidiation. Meanwhile, to assess a potential influence of the two treatments on H_2_O_2_ accumulation, DAB staining was carried out at 5 and 6 dpi. The rates of colonies with DAB stained conidiophore (s) were significantly lower in treated samples than the control (**Figures 7F**, **8B**). Similar results from another independent experiment are presented in Supplementary Figure S8. These data indicate that chelation of Ca^2+^ and antagonism of CaMs inhibit H_2_O_2_ generation and that both H_2_O_2_ and Ca^2+^ levels are tightly linked with asexual sporulation of *Bgt*.

### *B. graminis*-Specific Protein-Coding Genes

To identify proteins specific to *B. graminis* among the 685 proteins, we performed BLASTP alignments against the Nr database with an E-value cut off of 10^-5^. In total, 79 proteins without significant hits except from hits to other *Bgh* proteins were obtained (Supplementary Table S8). We did not find any annotations of these proteins in the COG, GO, KEGG and SwissProt databases. To gain more information, we aligned the 79 proteins against the *Bgh* protein database using BLASTP (E-value ≤ 1e-10). We found 47 putative effector proteins and 27 unknown hypothetical proteins. Among the 27 hypothetical genes, several genes were dramatically up-regulated during *Bgt* sporulation, for instance, two genes (EPQ64343.1, 18.5-fold rise in C5 dpi and EPQ62428.1, 13.3-fold rise in C4 dpi).

## Discussion

### Primary Metabolism in *B. graminis* during Conidiation

Asexual sporulation of *Bgt* is a complex process correlated with nutrient metabolism, energy consumption and fuel storage, which have been previously shown in other powdery mildews (Both et al., 2005; Wakefield et al., 2011). Our results indicate that besides glucose (reported previously; Both et al., 2005), other sugars, such as starch, sucrose, xylulose and fuculose, could serve as carbon sources for the foot cell and conidiophore development. This means that *B. graminis* may use specific sugars for conidiation like other fungi, for example, *Hypocrea atroviridis*, which has carbon-source predilection in conidiation (Friedl et al., 2008). It is well-known that energy is generated through glycolysis in cytosol and the TAC in mitochondria where acetyl-CoA is completely oxidized. Acetyl-CoA can be produced from the unsaturated fatty acid degradation and then enter the TAC so as to generate more energy. These results agree with the recent finding that deletion mutants of *PEX6* in *Coniothyrium minitans* cannot utilize oleic acid and produce considerably fewer conidia than the wild type (Wei et al., 2013). These results indicate that, compared with vegetative growth, *Bgt* needs more energy, which could come from glucose and acetyl-CoA oxidation, during conidiation. A major change in anabolism was conversion of glucose into glycogen during the conidiophore development. In addition, up-regulation of the caleosin protein-related gene occurred, which suggests that storage lipid bodies might be associated with *Bgt* conidiation. Glycogen and lipids are universally used for energy storage in fungal conidia. For example, glycogen and lipids have been detected in *Bgh* conidiophores as fuel for the next infection (Both et al., 2005). Caleosins are strongly correlated with lipid body maintenance and can be used as storage compounds in plant seed (Partridge and Murphy, 2009). Although diverse fatty acids have been detected in conidia of *B. graminis* (Muchembled et al., 2005), we have no direct evidence from the RNA-seq data that fatty acid biosynthesis is activated. This will need further studies focused on lipid and fatty acid biosynthesis during conidia formation and release.

### Comparison of the Genetic Control of Conidiation between *Bgt* and Other Fungi

It is known that several important signal pathways such as the BrlA pathway, G-protein signaling, ROS and Ca^2+^ mediated signaling have essential roles in coordinating conidiation in other fungi (Nguyen Q.B. et al., 2008; Park and Yu, 2012; Medina-Castellanos et al., 2014). Some orthologous genes in these pathways are differentially expressed during conidiation of powdery mildews, including *E. necator* (Wakefield et al., 2011). Our transcriptome analysis also identified some orthologs of the regulatory factors, such as the velvet complex components, that participate in these pathways. Therefore, the genetic control of conidiogenesis in *B. graminis* shares conserved elements known from other fungi. In addition, oxylipins, products produced from unsaturated fatty acid oxidization, have been known to function as signal molecules in fungal conidiation (Herrero-Garcia et al., 2011; Scala et al., 2014). Moreover, in biosynthesis of oxylipins, PpoA, one of the conserved fatty acid oxygenases plays a crucial role in regulating conidiation (Brown et al., 2009). Activation of the oxylipins biosynthesis-related gene in *Bgt* might suggest that the oxylipin pathway is also implicated in conidiogenesis of powdery mildews. Nevertheless, powdery mildews may recruit their unique proteins with unknown function as well, based on the result that several *B. graminis*-specific hypothetical genes were significantly up-regulated during conidiation. Functional characterization of these genes may contribute to uncovering unusual conidiogenesis mechanism employed by powdery mildews.

### Roles of H_2_O_2_ in Conidiogenesis

H_2_O_2_ has dual roles, acting as both a signal molecule and a stressor during *Bgt* asexual reproduction. Normal conidiation is correlated with a strong accumulation of H_2_O_2_, while impaired conidiation is associated with reduced H_2_O_2_ levels. This suggests that H_2_O_2_ is tightly linked with *Bgt* conidiogenesis. This observation is in accordance with the previous finding in *C. minitans* mutants involved in the disruption of the *CmPEX6* gene result in a significant decrease in the amount of H_2_O_2_, as well as reductions in conidia production compared to the values of the wild type (Wei et al., 2013). H_2_O_2_ also plays an important role in activating the oxidative stress response and triggering conidiation, partially via regulating the redox-responsive regulators (YAP1 and SKN7) and the HOG1 mediated pathway in *A. alternata* (Chung, 2012). In this pathway, YAP1 acts in H_2_O_2_ sensing and activation of antioxidants and the H_2_O_2_ signal can be transmitted through HOG1, which is regulated by NoxA and HSK. Comparatively, based on the expression profiles of the *NoxA*, the *HSK*, two *YAP1s* and the *HOG1* during *Bgt* conidiation, it seems that the H_2_O_2_ signal is likely transmitted through HOG1 as well. However, unlike the situation in *A. alternata*, the NoxA and the YAP1s in *Bgt* appear not to sense and respond to H_2_O_2_ and the NoxA and the HSK seem not to be associated with transcriptional activation of *HOG1*. Further functional analysis of these genes will test this hypothesis. It appears, however, that the concentration of cellular H_2_O_2_ must be strictly regulated in time and space, since excessive H_2_O_2_ is undoubtedly harmful to the membrane system of organelles in cells (Sies, 2014). According to our data, the activated antioxidant system provided a ‘redox-buffer’ to detoxify H_2_O_2_ so as to modulate increased H_2_O_2_ level, and thereby enhance resistance to this oxidative stress. This result suggests the importance of maintaining redox homeostasis during *Bgt* conidiation. This balance is also required during interaction between *Botrytis cinerea* and its host to ensure normal growth and virulence (Viefhues et al., 2014).

### Source of H_2_O_2_

We propose two possible explanations for the source of H_2_O_2_ accumulated in the developing conidiophores. One is uptake from host epidermis cells by haustoria, and the other is production of H_2_O_2_ by the pathogen itself. Because the H_2_O_2_ burst was obviously suppressed in the infected cells of Chancellor (**Figure 4B**), it is unlikely that most of it was derived from the host. For producing by itself, O^2-^ can be generated through the metabolic reactions catalyzed by the Nox complex on the plasma membrane and then can be further dismutated to H_2_O_2_ by superoxide dismutases (Toledano et al., 2010; Reddi and Culotta, 2013). Another source of O^2-^ is mitochondria, from which it can be generated via the ETC and released (Suraniti et al., 2014). Note that we found that the ETC was enhanced, while the *NoxA* gene expression was repressed at the foot cell forming stage. We speculate that more H_2_O_2_ is produced by the pathogen itself through mitochondria. This is supported by the findings that enhanced activity of the ETC gives rise to generate more O^2-^ (Liu et al., 2016), while inhibition of Ca^2+^ signaling, which plays a positive role in mitochondrial ATP synthesis (Feissner et al., 2009), markedly affects H_2_O_2_ accumulation in our investigation.

### Involvement of Ca^2+^, H_2_O_2_, and PIP Mediated Signaling Pathways in Conidiogenesis

A crosslink between Ca^2+^ and H_2_O_2_ mediated signaling pathways is present in *Bgt* conidiogenesis. One of the most important results of this study is the finding that disturbed Ca^2+^/CaM signaling have a negative influence on both H_2_O_2_ production and asexual reproduction of the pathogen. An alternative explanation is that the treatments with the two drugs altered the defense state of the host, including pre- and post-invasion defenses (Lipka et al., 2005), which caused less conidiation. However, 5 mM EGTA and 12.5 μM TFP did not affect the haustorium formation of the pathogen significantly. This means that the preinvasion resistance is not induced. Furthermore, according to the results from DAB staining, very little H_2_O_2_ was detected in the infected epidemic cells (**Figure 8**). This indicates that the post-invasion defense responses during the interaction between the pathogen and the host at 5 and 6 dpi are suppressed. Therefore, the significant reduction in conidiation does not result from the alteration of defense state of the host. These data suggest that the two pathways have crucial roles in regulating conidiation of *Bgt* and a communication between them is present. The crosslink between Ca^2+^ and H_2_O_2_ signaling has been reported in mammals (Feissner et al., 2009; Liu et al., 2016), plant (Gonzalez et al., 2012) and fungi, such as *Trichoderma atroviride* (Medina-Castellanos et al., 2014). The HOG1 with a putative role in ROS signaling showed a significant sequence similarity to the CCaMKs in *Bgh*, which suggests that it may have dual functions in the crosslinked signaling mediated by H_2_O_2_ and Ca^2+^. In addition, since the antioxidant protein coding gene expressions can be induced by CCaMKs in plants (Gonzalez et al., 2012; Ma et al., 2012), those in *Bgt* are possibly induced by the HOG1 during conidiation. For initiation of Ca^2+^ fluxes, it can be activated through Ca^2+^ channels or IP3 sensitive channels (Wen et al., 2015). However, no significant differential expression of genes involved in Ca^2+^ channels or exchangers was detected. Alternatively, activated PIP signaling can lead to release of IP3, which can induce Ca^2+^ fluxes from endoplasmic reticulum to cytosol (Alzayady et al., 2016). Hence, PIP signaling is likely involved in the release of free Ca^2+^. More evidence from monitoring of cellular free Ca^2+^ and IP3 levels is needed to confirm this hypothesis. Taken together, our data indicate that at least three signaling pathways mediated by Ca^2+^, H_2_O_2_, and PIP are active in regulating conidiogenesis of *B. graminis*.

## Conclusion

Several events in primary metabolism including the activation of metabolism of diverse sugars, glycogen synthesis, energy production, and unsaturated fatty acid oxidation occurred during *Bgt* conidiation. A crosslink between H_2_O_2_ and Ca^2+^ signaling and involvement of some other regulators are associated with regulation of *Bgt* conidiation. Our findings broadened and strengthened our knowledge of conidiogenesis of powdery mildews.

## Author Contributions

F-SZ and D-ZY conceived and designed the research. F-SZ, X-JZ, S-JG, W-QS, and L-JY contributed to the development of material. F-SZ, FM, and M-FX analyzed the data. F-SZ and S-JG performed the pharmacological experiments and histological investigation. F-SZ wrote the manuscript. FM and D-ZY edited the manuscript.

## Conflict of Interest Statement

The authors declare that the research was conducted in the absence of any commercial or financial relationships that could be construed as a potential conflict of interest.

## Footnotes

1 http://www.blast2go.com

2 http://www.bioconductor.org/packages/release/bioc/html/topGO.html

3 http://www.genome.jp/kegg/

4 www.cbs.dtu.dk/services/TMHMM/

5 http://ftfd.snu.ac.kr/index.php?a=view

## References

[B1] Alkhayyat F. Chang Kim S. Yu J. H. (2015). Genetic control of asexual development in *Aspergillus fumigatus*. *Adv. Appl. Microbiol.* 90 93–107. 10.1016/bs.aambs.2014.09.004 25596030

[B2] Alzayady K. J. Wang L. Chandrasekhar R. Wagner L. E. Van Petegem F. Yule D. I. (2016). Defining the stoichiometry of inositol 1,4,5-trisphosphate binding required to initiate Ca2+ release. *Sci. Signal.* 9:ra35 10.1126/scisignal.aad6281 PMC4850551 27048566

[B3] Anders S. Huber W. (2010). Differential expression analysis for sequence count data. *Genome Biol.* 11:R106 10.1186/gb-2010-11-10-r106 PMC3218662 20979621

[B4] Bindschedler L. V. Panstruga R. Spanu P. D. (2016). Mildew-omics: how global analyses aid the understanding of Life and evolution of powdery mildews. *Front. Plant Sci.* 7:123 10.3389/fpls.2016.00123 PMC4753294 26913042

[B5] Both M. Csukai M. Stumpf M. P. Spanu P. D. (2005). Gene expression profiles of *Blumeria graminis* indicate dynamic changes to primary metabolism during development of an obligate biotrophic pathogen. *Plant Cell* 17 2107–2122. 10.1105/tpc.105.032631 15951491 PMC1167555

[B6] Briggle L. W. (1969). Near-isogenic lines of wheat with genes for resistance to *Erysiphe graminis* f. sp. *tritici*. *Crop Sci.* 9 70–72. 10.2135/cropsci1969.0011183X000900010023x

[B7] Brown J. K. Hovmoller M. S. (2002). Aerial dispersal of pathogens on the global and continental scales and its impact on plant disease. *Science* 297 537–541. 10.1126/science.1072678 12142520

[B8] Brown S. H. Scott J. B. Bhaheetharan J. Sharpee W. C. Milde L. Wilson R. A. (2009). Oxygenase coordination is required for morphological transition and the host-fungus interaction of *Aspergillus flavus*. *Mol. Plant Microbe Interact.* 22 882–894. 10.1094/mpmi-22-7-0882 19522570

[B9] Chen X. Xu C. Qian Y. Liu R. Zhang Q. Zeng G. (2016). MAPK cascade-mediated regulation of pathogenicity, conidiation and tolerance to abiotic stresses in the entomopathogenic fungus *Metarhizium robertsii*. *Environ. Microbiol.* 18 1048–1062. 10.1111/1462-2920.13198 26714892

[B10] Chung K. R. (2012). Stress response and pathogenicity of the necrotrophic fungal pathogen *Alternaria alternata*. *Scientifica* 2012:635431 10.6064/2012/635431 PMC3820455 24278721

[B11] Dean R. Van Kan J. A. Pretorius Z. A. Hammond-Kosack K. E. Di Pietro A. Spanu P. D. (2012). The top 10 fungal pathogens in molecular plant pathology. *Mol. Plant Pathol.* 13 414–430. 10.1111/j.1364-3703.2011.00783.x 22471698 PMC6638784

[B12] Feissner R. F. Skalska J. Gaum W. E. Sheu S. S. (2009). Crosstalk signaling between mitochondrial Ca2+ and ROS. *Front. Biosci.* 14 1197–1218. 10.2741/3303 PMC2683671 19273125

[B13] Friedl M. A. Kubicek C. P. Druzhinina I. S. (2008). Carbon source dependence and photostimulation of conidiation in *Hypocrea atroviridis*. *Appl. Environ. Microbiol.* 74 245–250. 10.1128/aem.02068-07 17981948 PMC2223218

[B14] Gadoury D. M. Wakefield L. M. Cadle-Davidson L. Dry I. B. Seem R. C. (2012). Effects of prior vegetative growth, inoculum density, light, and mating on conidiation of *Erysiphe necator*. *Phytopathology* 102 65–72. 10.1094/PHYTO-03-11-0085 21848394

[B15] Glawe D. A. (2008). The powdery mildews: a review of the world’s most familiar (yet poorly known) plant pathogens. *Annu. Rev. Phytopathol.* 46 27–51. 10.1146/annurev.phyto.46.081407.104740 18680422

[B16] Gonzalez A. de ios Angeles Cabrera M. Henriquez M. J. Contreras R. A. Morales B. Moenne A. (2012). Cross talk among calcium, hydrogen peroxide, and nitric oxide and activation of gene expression involving calmodulins and calcium-dependent protein kinases in *Ulva compressa* exposed to copper excess. *Plant Physiol.* 158 1451–1462. 10.1104/pp.111.191759 22234999 PMC3291273

[B17] Gotz S. Garcia-Gomez J. M. Terol J. Williams T. D. Nagaraj S. H. Nueda M. J. (2008). High-throughput functional annotation and data mining with the Blast2GO suite. *Nucleic Acids Res.* 36 3420–3435. 10.1093/nar/gkn176 18445632 PMC2425479

[B18] Guan Y. Wang D. Y. Ying S. H. Feng M. G. (2015). A novel Ras GTPase (Ras3) regulates conidiation, multi-stress tolerance and virulence by acting upstream of Hog1 signaling pathway in *Beauveria bassiana*. *Fungal Genet. Biol.* 82 85–94. 10.1016/j.fgb.2015.07.002 26162967

[B19] Hacquard S. Kracher B. Maekawa T. Vernaldi S. Schulze-Lefert P. van Themaat E. V. L. (2013). Mosaic genome structure of the barley powdery mildew pathogen and conservation of transcriptional programs in divergent hosts. *Proc. Natl. Acad. Sci. U.S.A.* 110 E2219–E2228. 10.1073/pnas.1306807110 23696672 PMC3683789

[B20] Herrero-Garcia E. Garzia A. Cordobés S. Espeso E. A. Ugalde U. (2011). 8-Carbon oxylipins inhibit germination and growth, and stimulate aerial conidiation in *Aspergillus nidulans*. *Fungal Biol.* 115 393–400. 10.1016/j.funbio.2011.02.005 21530921

[B21] Huang S. He Z. Zhang S. Keyhani N. O. Song Y. Yang Z. (2015). Interplay between calcineurin and the Slt2 MAP-kinase in mediating cell wall integrity, conidiation and virulence in the insect fungal pathogen *Beauveria bassiana*. *Fungal Genet. Biol.* 83 78–91. 10.1016/j.fgb.2015.08.009 26319315

[B22] Jørgensen J. H. Torp J. (1978). Distribution of spring barley varieties with different powdery mildew resistances in Denmark from 1960 to 1976. *R. Vet. Agric. Univ. YB.* 27–44.

[B23] Kim D. Pertea G. Trapnell C. Pimentel H. Kelley R. Salzberg S. L. (2013). TopHat2: accurate alignment of transcriptomes in the presence of insertions, deletions and gene fusions. *Genome Biol.* 14:R36 10.1186/gb-2013-14-4-r36 PMC4053844 23618408

[B24] Kusch S. Ahmadinejad N. Panstruga R. Kuhn H. (2014). In silico analysis of the core signaling proteome from the barley powdery mildew pathogen (*Blumeria graminis* f. sp. *hordei*). *BMC Genomics* 15:843 10.1186/1471-2164-15-843 PMC4195978 25277210

[B25] Li F. Wang Z. L. Zhang L. B. Ying S. H. Feng M. G. (2015). The role of three calcineurin subunits and a related transcription factor (Crz1) in conidiation, multistress tolerance and virulence in *Beauveria bassiana*. *Appl. Microbiol. Biotechnol.* 99 827–840. 10.1007/s00253-014-6124-6 25324131

[B26] Lipka V. Dittgen J. Bednarek P. Bhat R. Wiermer M. Stein M. (2005). Pre- and postinvasion defenses both contribute to nonhost resistance in *Arabidopsis*. *Science* 310 1180–1183. 10.1126/science.1119409 16293760

[B27] Liu C. Ye Y. Zhou Q. Zhang R. Zhang H. Liu W. (2016). Crosstalk between Ca2+ signaling and mitochondrial H2O2 is required for rotenone inhibition of mTOR signaling pathway leading to neuronal apoptosis. *Oncotarget* 7 7534–7549. 10.18632/oncotarget.7183 26859572 PMC4884936

[B28] Livak K. J. Schmittgen T. D. (2001). Analysis of relative gene expression data using real-time quantitative PCR and the 2(-ΔΔC(T)) method. *Methods* 25 402–408. 10.1006/meth.2001.1262 11846609

[B29] Ma F. Lu R. Liu H. Shi B. Zhang J. Tan M. (2012). Nitric oxide-activated calcium/calmodulin-dependent protein kinase regulates the abscisic acid-induced antioxidant defence in maize. *J. Exp. Bot.* 63 4835–4847. 10.1093/jxb/ers161 22865912 PMC3427994

[B30] Medina-Castellanos E. Esquivel-Naranjo E. Heil M. Herrera-Estrella A. (2014). Extracellular ATP activates MAPK and ROS signaling during injury response in the fungus *Trichoderma atroviride*. *Front. Plant Sci.* 5:659 10.3389/fpls.2014.00659 PMC4240048 25484887

[B31] Moriura N. Matsuda Y. Oichi W. Nakashima S. Hirai T. Sameshima T. (2006). Consecutive monitoring of lifelong production of conidia by individual conidiophores of *Blumeria graminis* f. sp. *hordei* on barley leaves by digital microscopic techniques with electrostatic micromanipulation. *Mycol. Res.* 110(Pt 1), 18–27. 10.1016/j.mycres.2005.09.007 16378716

[B32] Muchembled J. Sahraoui A. L. Laruelle F. Palhol F. Couturier D. Grandmougin-Ferjani A. (2005). Methoxylated fatty acids in Blumeria graminis conidia. *Phytochemistry* 66 793–796. 10.1016/j.phytochem.2005.02.011 15797605

[B33] Nguyen Q. B. Kadotani N. Kasahara S. Tosa Y. Mayama S. Nakayashiki H. (2008). Systematic functional analysis of calcium-signalling proteins in the genome of the rice-blast fungus, *Magnaporthe oryzae*, using a high-throughput RNA-silencing system. *Mol. Microbiol.* 68 1348–1365. 10.1111/j.1365-2958.2008.06242.x 18433453

[B34] Nguyen S. D. Baes M. Van Veldhoven P. P. (2008). Degradation of very long chain dicarboxylic polyunsaturated fatty acids in mouse hepatocytes, a peroxisomal process. *Biochim. Biophys. Acta* 1781 400–405. 10.1016/j.bbalip.2008.06.004 18619556

[B35] Park H. S. Yu J. H. (2012). Genetic control of asexual sporulation in filamentous fungi. *Curr. Opin. Microbiol.* 15 669–677. 10.1016/j.mib.2012.09.006 23092920

[B36] Park J. Park J. Jang S. Kim S. Kong S. Choi J. (2008). FTFD: an informatics pipeline supporting phylogenomic analysis of fungal transcription factors. *Bioinformatics* 24 1024–1025. 10.1093/bioinformatics/btn058 18304934

[B37] Partridge M. Murphy D. J. (2009). Roles of a membrane-bound caleosin and putative peroxygenase in biotic and abiotic stress responses in *Arabidopsis*. *Plant Physiol. Biochem.* 47 796–806. 10.1016/j.plaphy.2009.04.005 19467604

[B38] Reddi A. R. Culotta V. C. (2013). SOD1 integrates signals from oxygen and glucose to repress respiration. *Cell* 152 224–235. 10.1016/j.cell.2012.11.046 23332757 PMC3552299

[B39] Scala V. Giorni P. Cirlini M. Ludovici M. Visentin I. Cardinale F. (2014). LDS1-produced oxylipins are negative regulators of growth, conidiation and fumonisin synthesis in the fungal maize pathogen *Fusarium verticillioides*. *Front. Microbiol.* 5:669 10.3389/fmicb.2014.00669 PMC4263177 25566199

[B40] Schulze S. K. Kanwar R. Gölzenleuchter M. Therneau T. M. Beutler A. S. (2012). SERE: single-parameter quality control and sample comparison for RNA-Seq. *BMC Genomics* 13:524 10.1186/1471-2164-13-524 PMC3534338 23033915

[B41] Sies H. (2014). Role of metabolic H2O2 generation: redox signaling and oxidative stress. *J. Biol. Chem.* 289 8735–8741. 10.1074/jbc.R113.544635 24515117 PMC3979367

[B42] Spanu P. D. Abbott J. C. Amselem J. Burgis T. A. Soanes D. M. Stuber K. (2010). Genome expansion and gene loss in powdery mildew fungi reveal tradeoffs in extreme parasitism. *Science* 330 1543–1546. 10.1126/science.1194573 21148392

[B43] Suraniti E. Ben-Amor S. Landry P. Rigoulet M. Fontaine E. Bottari S. (2014). Electrochemical monitoring of the early events of hydrogen peroxide production by mitochondria. *Angew. Chem. Int. Ed. Engl.* 53 6655–6658. 10.1002/anie.201403096 24854602

[B44] Tatusov R. L. Koonin E. V. Lipman D. J. (1997). A genomic perspective on protein families. *Science* 278 631–637. 10.1126/science.278.5338.631 9381173

[B45] Thordal-Christensen H. Zhang Z. Wei Y. Collinge D. B. (1997). Subcellular localization of H2O2 in plants. H2O2 accumulation in papillae and hypersensitive response during the barley-powdery mildew interaction. *Plant J.* 11 1187–1194. 10.1046/j.1365-313X.1997.11061187.x

[B46] Toledano M. B. Planson A. G. Delaunay-Moisan A. (2010). Reining in H2O2 for safe signaling. *Cell* 140 454–456. 10.1016/j.cell.2010.02.003 20178737

[B47] Trapnell C. Williams B. A. Pertea G. Mortazavi A. Kwan G. van Baren M. J. (2010). Transcript assembly and quantification by RNA-Seq reveals unannotated transcripts and isoform switching during cell differentiation. *Nat. Biotechnol.* 28 511–515. 10.1038/nbt.1621 20436464 PMC3146043

[B48] van Rossum H. M. Kozak B. U. Pronk J. T. van Maris A. J. (2016). Engineering cytosolic acetyl-coenzyme a supply in *Saccharomyces cerevisiae*: pathway stoichiometry, free-energy conservation and redox-cofactor balancing. *Metab. Eng.* 36 99–115. 10.1016/j.ymben.2016.03.006 27016336

[B49] Viefhues A. Heller J. Temme N. Tudzynski P. (2014). Redox systems in *Botrytis cinerea*: impact on development and virulence. *Mol. Plant Microbe Interact.* 27 858–874. 10.1094/mpmi-01-14-0012-r 24983673

[B50] Wakefield L. Gadoury D. M. Seem R. C. Milgroom M. G. Sun Q. Cadle-Davidson L. (2011). Differential gene expression during conidiation in the grape powdery mildew pathogen, *Erysiphe necator*. *Phytopathology* 101 839–846. 10.1094/PHYTO-11-10-0295 21405992

[B51] Wanders R. J. Waterham H. R. (2006). Biochemistry of mammalian peroxisomes revisited. *Annu. Rev. Biochem.* 75 295–332. 10.1146/annurev.biochem.74.082803.133329 16756494

[B52] Wang Y. Geng Z. Jiang D. Long F. Zhao Y. Su H. (2013). Characterizations and functions of regulator of G protein signaling (RGS) in fungi. *Appl. Microbiol. Biotechnol.* 97 7977–7987. 10.1007/s00253-013-5133-1 23917634

[B53] Wei W. Zhu W. Cheng J. Xie J. Jiang D. Li G. (2016). Nox Complex signal and MAPK cascade pathway are cross-linked and essential for pathogenicity and conidiation of mycoparasite *Coniothyrium minitans*. *Sci. Rep.* 6:24325 10.1038/srep24325 PMC4828707 27066837

[B54] Wei W. Zhu W. Cheng J. Xie J. Li B. Jiang D. (2013). CmPEX6, a gene involved in peroxisome biogenesis, is essential for parasitism and conidiation by the sclerotial parasite *Coniothyrium minitans*. *Appl. Environ. Microbiol.* 79 3658–3666. 10.1128/aem.00375-13 23563946 PMC3675954

[B55] Wen H. Xu W. J. Jin X. Oh S. Phan C. H. Song J. (2015). The roles of IP3 receptor in energy metabolic pathways and reactive oxygen species homeostasis revealed by metabolomic and biochemical studies. *Biochim. Biophys. Acta* 1853(11 Pt A), 2937–2944. 10.1016/j.bbamcr.2015.07.020 26235438

[B56] Wicker T. Oberhaensli S. Parlange F. Buchmann J. P. Shatalina M. Roffler S. (2013). The wheat powdery mildew genome shows the unique evolution of an obligate biotroph. *Nat. Genet.* 45 1092–1096. 10.1038/ng.2704 23852167

[B57] Yang X. Yang L. Yu D. Ni H. (2008). Effects of physcion, a natural anthraquinone derivative, on the infection process of *Blumeria graminis* on wheat. *Can. J. Plant Pathol.* 30 391–396. 10.1080/07060660809507536

[B58] Yu J. H. (2006). Heterotrimeric G protein signaling and RGSs in *Aspergillus nidulans*. *J. Microbiol.* 44 145–154. 16728950

[B59] Zeng F. Yang L. Gong S. Shi W. Zhang X. Wang H. (2014). Virulence and diversity of *Blumeria graminis* f. sp. *tritici* populations in China. *J. Integr. Agric.* 13 2424–2437. 10.1016/S2095-3119(13)60669-3

[B60] Zhang Y. Zagnitko O. Rodionova I. Osterman A. Godzik A. (2011). The FGGY carbohydrate kinase family: insights into the evolution of functional specificities. *PLoS Comput. Biol.* 7:e1002318 10.1371/journal.pcbi.1002318 PMC3245297 22215998

