# Supplementary material for: Transcriptome Analyses Shed New Insights into Primary Metabolism and Regulation of Blumeria graminis f. sp. tritici during Conidiation
Source: Front Plant Sci. 2017 Jun 30;8:1146. doi: 10.3389/fpls.2017.01146 (PMC5492466; doi:10.3389/fpls.2017.01146)
Supplement: Supplementary file 9 [file Presentation_1.PDF]

Supplementary Material:

Transcriptome analyses shed new insights into primary metabolism and regulation of *Blumeria graminis* f. sp. *tritici* during conidiation

Fan-Song Zeng[1,2,3], Fabrizio Menardo[4], Min-Feng Xue[2,3], Xue-Jiang Zhang[2,3], Shuang-Jun Gong[2,3], Li-Jun Yang[1,2,3], Wen-Qi Shi[2,3] & Da-Zhao Yu[1,2,3*]

[1] College of Life Science, Wuhan University, Wuhan, P. R. China

[2] Key Laboratory of Integrated Pest Management on Crops in Central China, Ministry of Agriculture, Wuhan, P. R. China

[3]Institute of Plant Protection and Soil Science, Hubei Academy of Agricultural Sciences, Wuhan, P. R. China

[4] Institute of Plant and Microbial Biology, University of Zürich, Zürich, Switzerland

*** Correspondence:**

Author Name: Da-Zhao Yu

E-mail: dazhaoyu@china.com

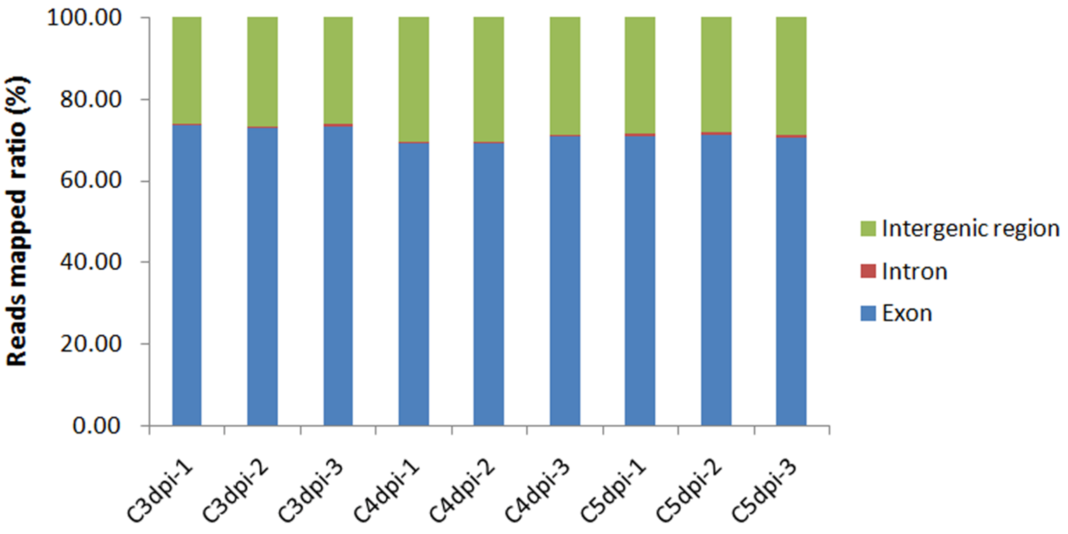

Fig. S1 Reads mapped ratio of nine cDNA libraries to the reference genome of *Blumeria. graminis* f. sp. *tritici* (*Bgt*) isolate 96224. More than 65% reads of the combined data were mapped to the exon region and the rest were mapped to the intergenic region and sequence of the intron of the coding genes.

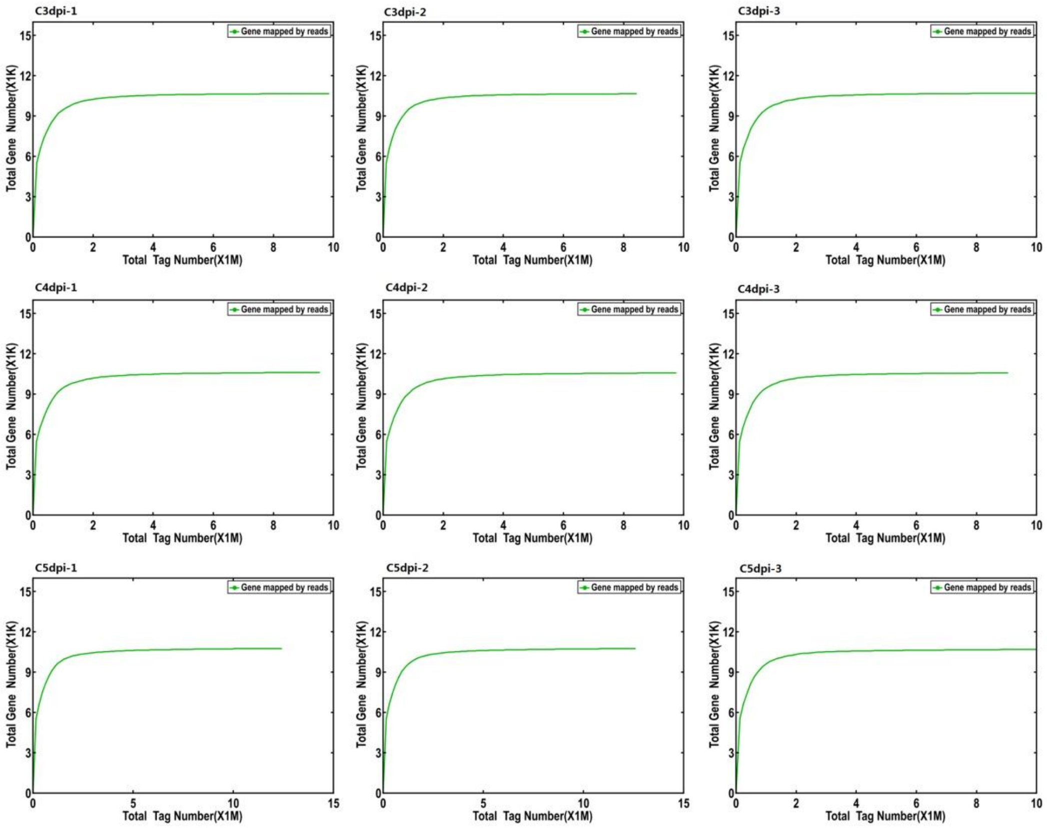

Fig. S2 Saturation analysis for the sequencing depth of nine cDNA libraries. The number of detected genes ceases to increase when the number of reads reaches 10M. These results indicate that the nine libraries were sequenced to saturation.

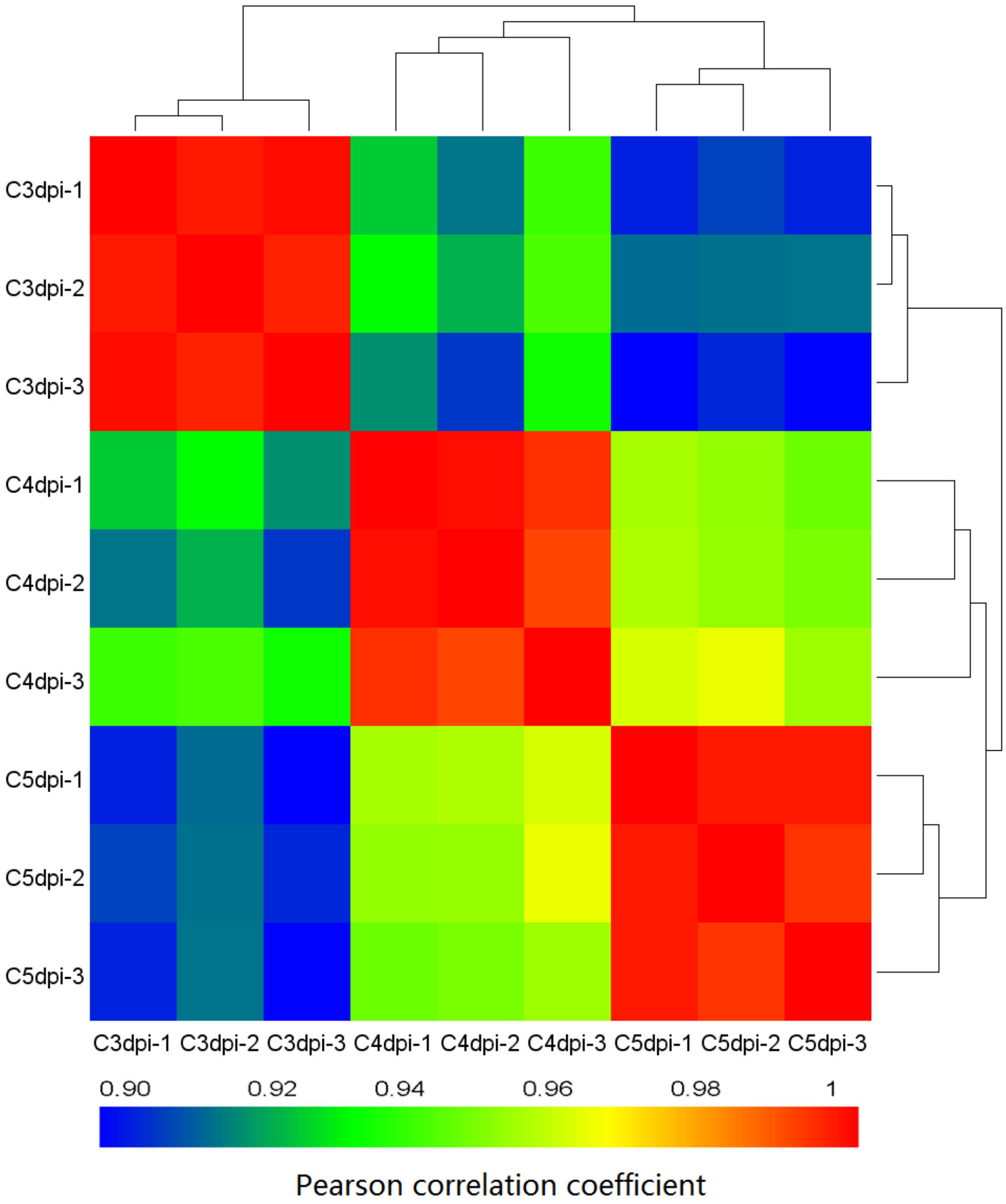

Fig. S3 Correlation matrix of *Bgt* expression profiles during conidiation. The Pearson correlation coefficients (r^2) of gene FPKM between replicates were calculated using the method of Simple Error Ratio Estimate (SERE, see material and method).

(A)

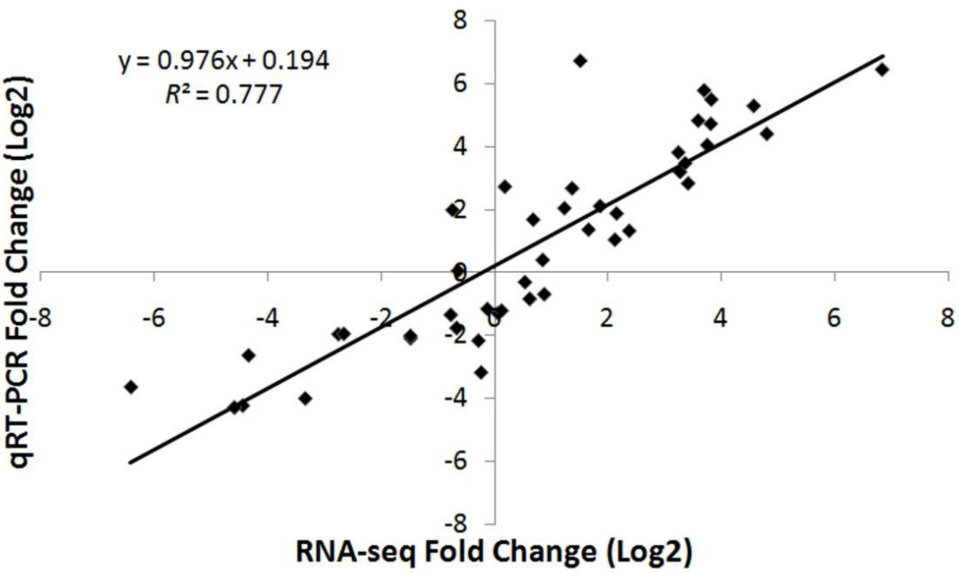
y = 0.976x + 0.194
R² = 0.777
qRT-PCR Fold Change (Log2)
RNA-seq Fold Change (Log2)
-8
-6
-4
-2
0
2
4
6
8

(B)

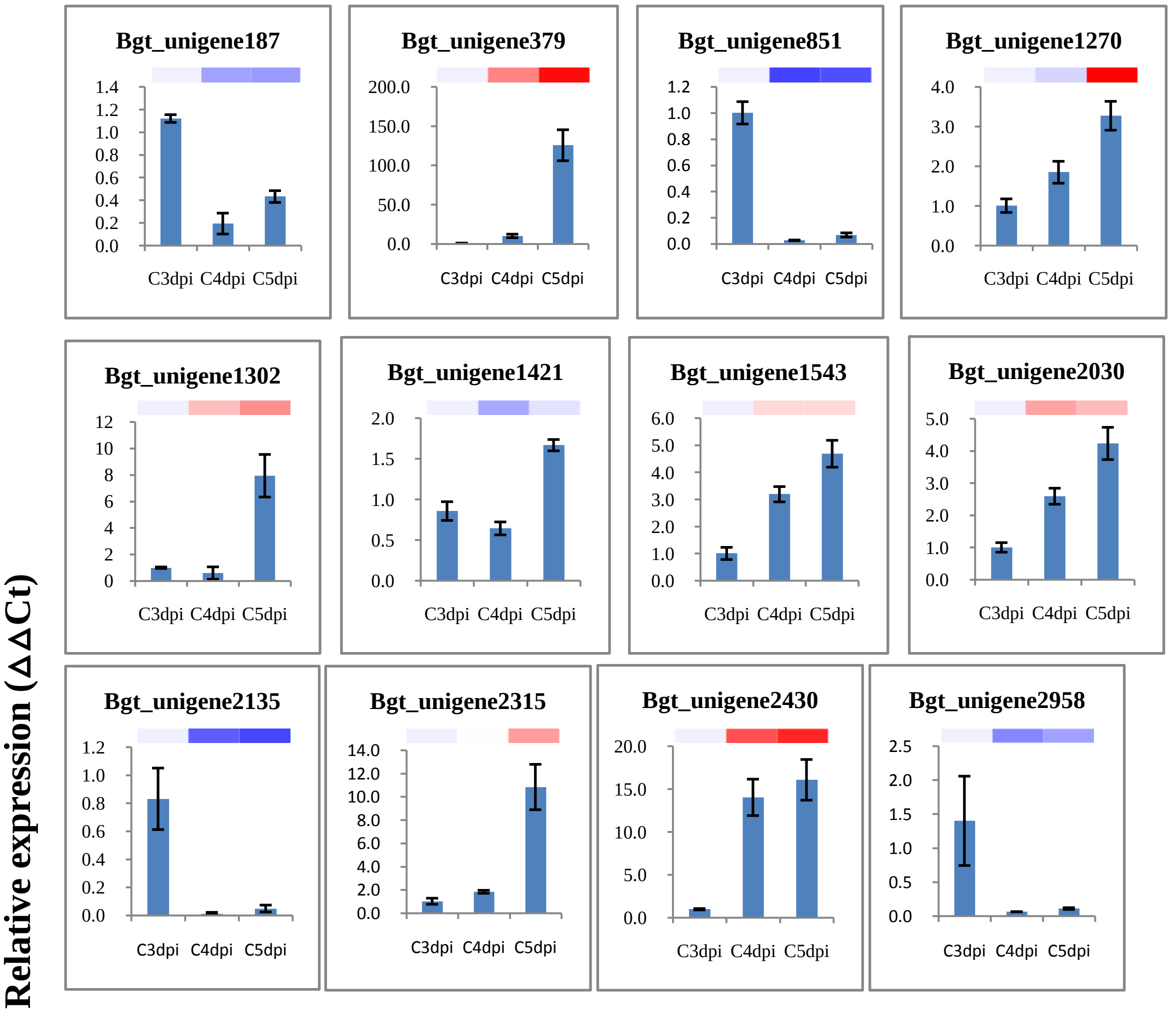
Relative expression (ΔΔCt)
Bgt_unigene187
Bgt_unigene379
Bgt_unigene851
Bgt_unigene1270
Bgt_unigene1302
Bgt_unigene1421
Bgt_unigene1543
Bgt_unigene2030
Bgt_unigene2135
Bgt_unigene2315
Bgt_unigene2430
Bgt_unigene2958
C3dpi C4dpi C5dpi

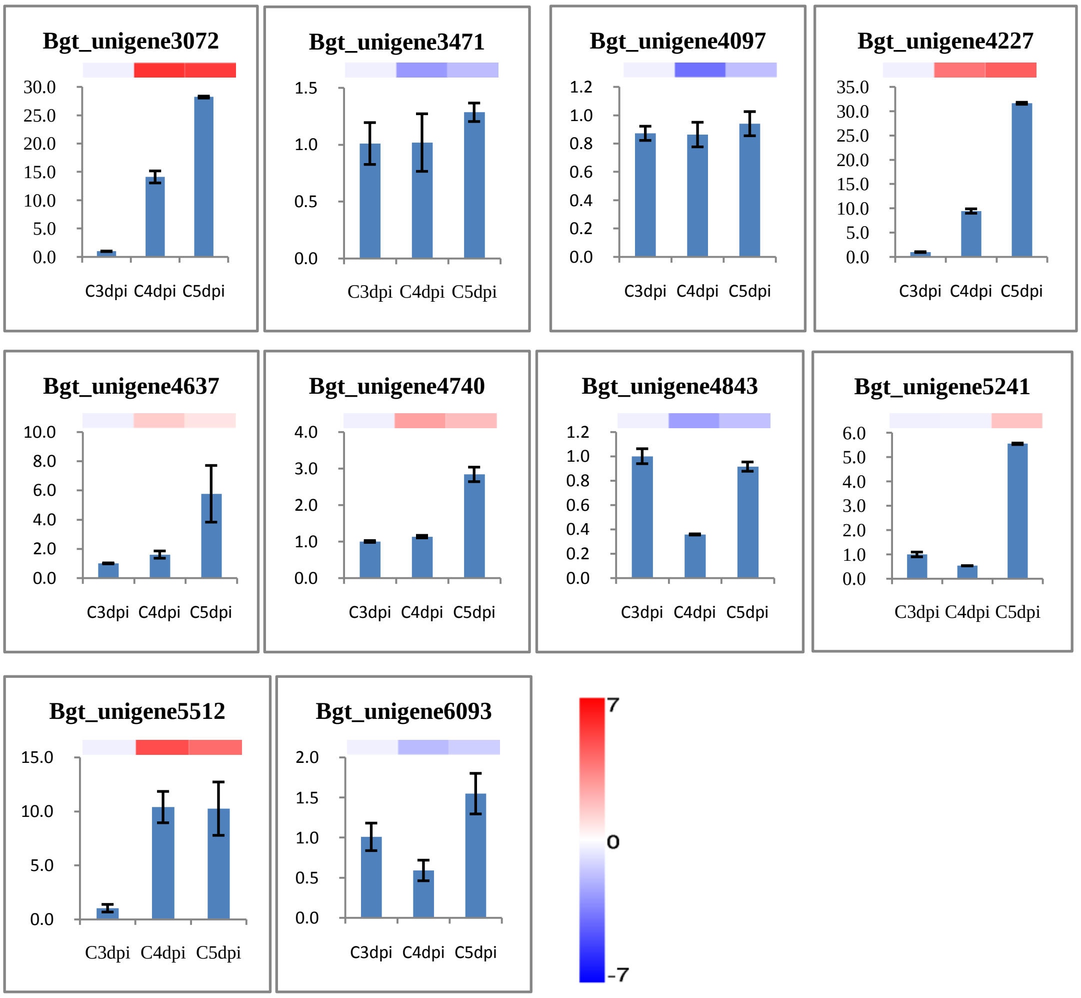

Fig. S4 Validation of RNA-seq data by real-time quantitative RT-PCR. Twenty-two differentially expressed genes (DEGs) were selected and real-time quantitative RT-PCR was conducted to verify the quality of expression changes in RNA-seq (log2 fold). Results are shown by the scatter plot for genes differentially expressed in the pairs of C4dpi/C3dpi and C5dpi/C3dpi (A) and bar graphs for the expression profiles of individual gene (B). Color panels above the bars represent log2-transformed mean expression values obtained by RNA-seq (see reference color bar).

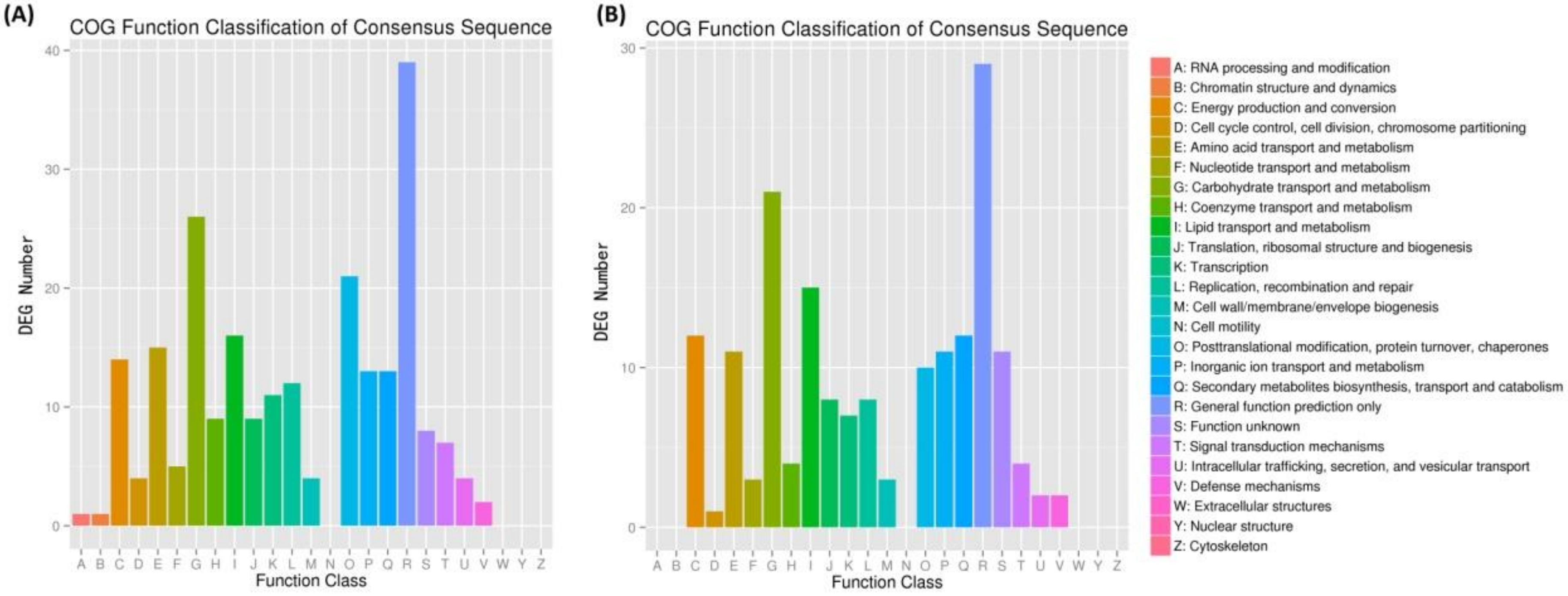

Fig. S5 Functional classification in the COG database of 685genes differentially expressed during *Bgt* conidiation. Totally, 181 out of 556 DEGs from the comparison of C4dpi/C3dpi were annotated (A); 128 out of 404 DEGs from the comparison of C5dpi/C3dpi were annotated (B).

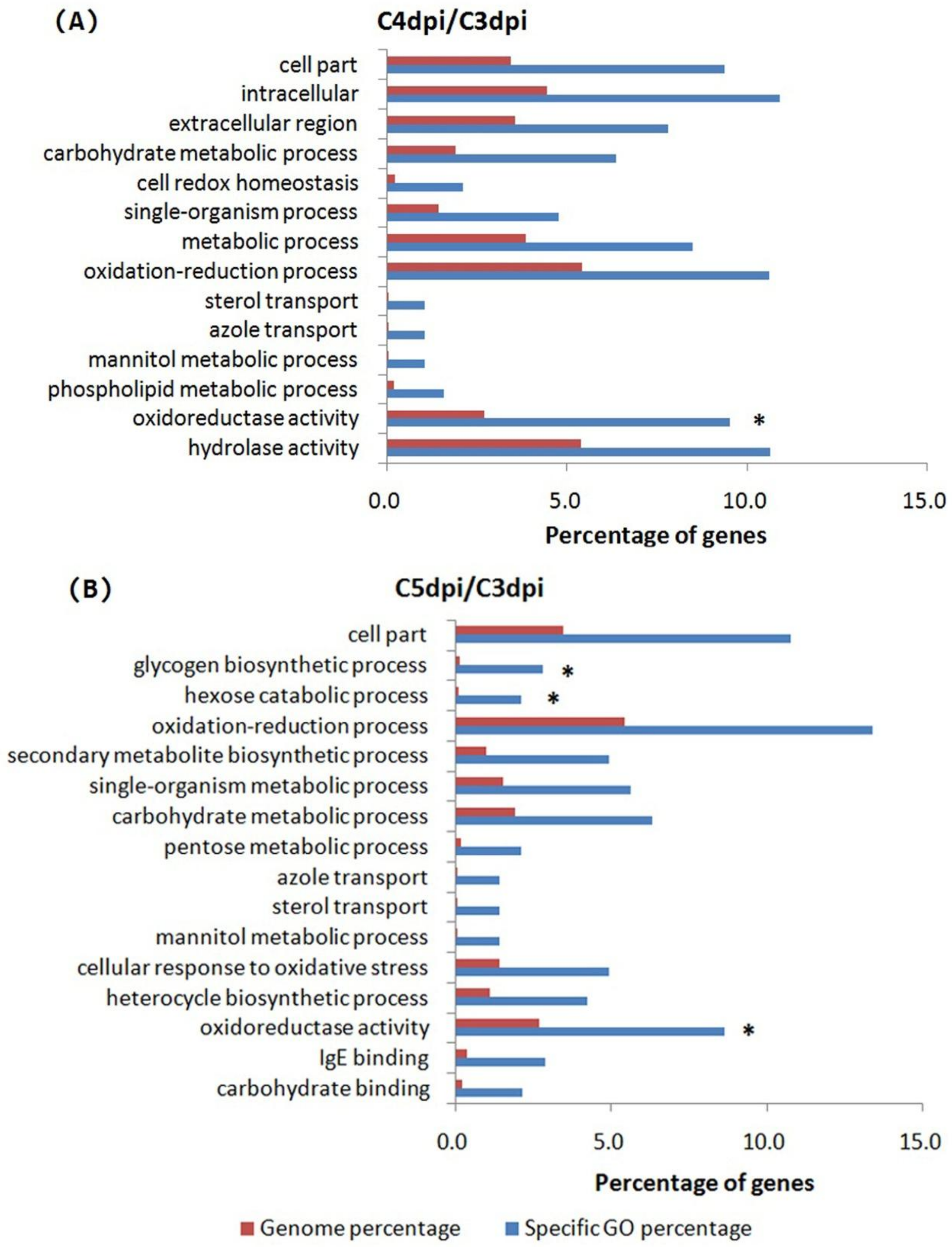

Fig. S6 Enriched gene ontology (GO) terms from genes differentially expressed during asexual development of *Bgt*. Fourteen and sixteen GO terms in the pair-wise comparisons of C4dpi/C3dpi (A) and C5dpi/C3dpi (B) with non-corrected *p*-values <0.005 based on Fisher's exact test were shown, respectively. Of them, three terms were significantly enriched with false discovery rate (FDR) corrected *p*-values <0.05 (indicated with asterisk).

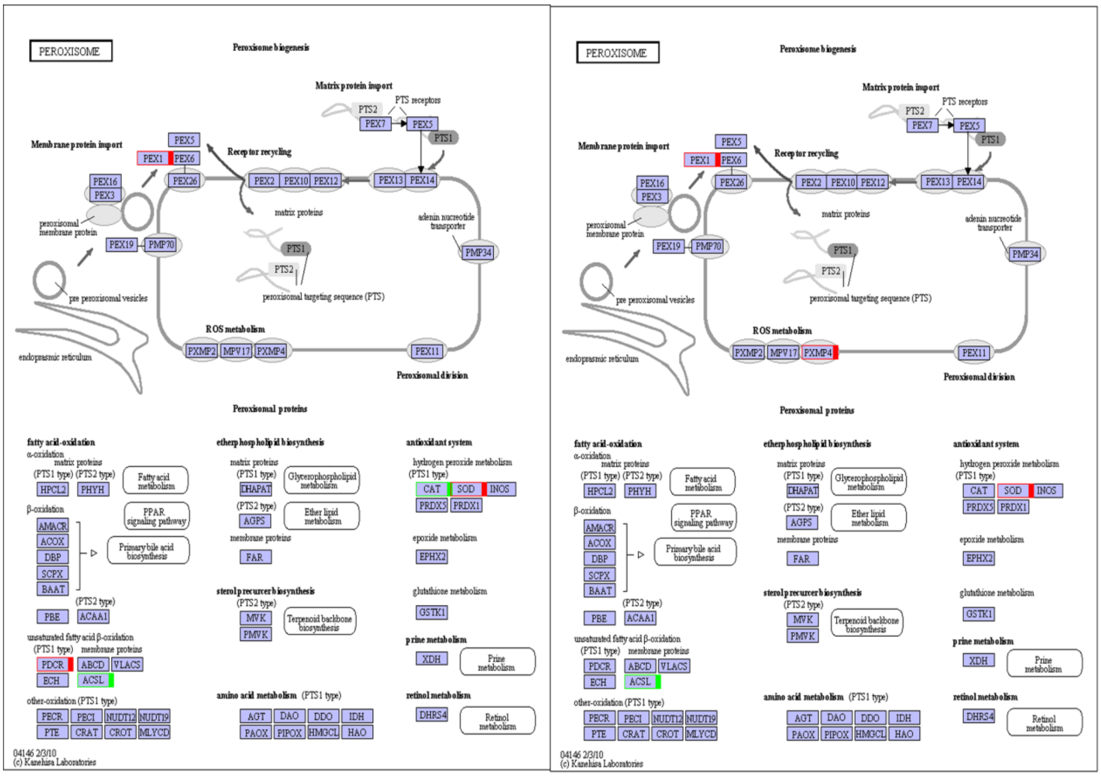

C4dpi/C3dpi C5dpi/C3dpi

Fig. S7 DEGs matched with enzymes in the KEGG metabolic pathway of "peroxisome" (ko04146) during asexual sporulation of *Bgt*. Red indicate that the DEGs encoding corresponding enzyme were up-regulated and green represent down-regulation.

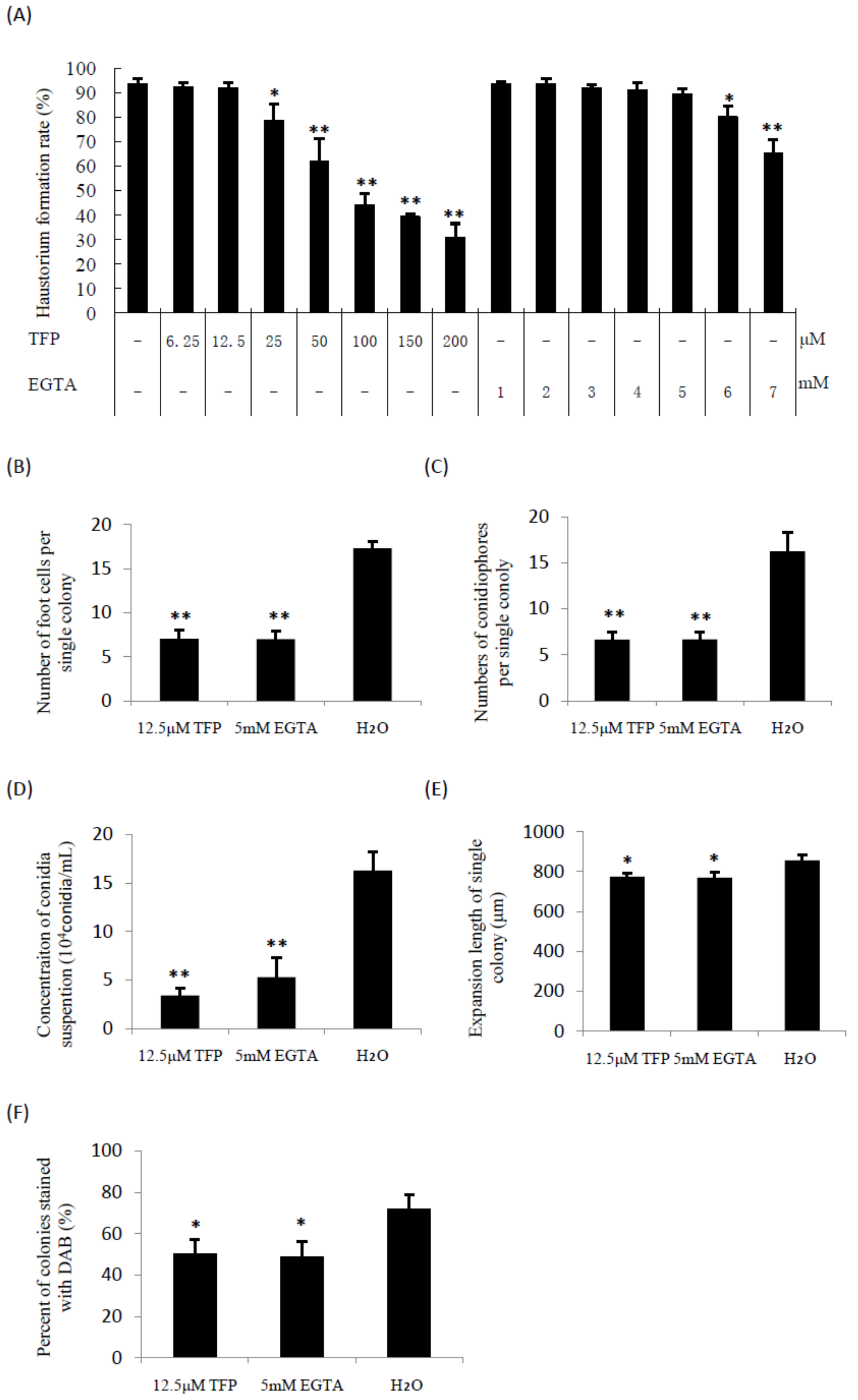

Fig. S8. Data from another independent experiment showing similar results to the data

represented in Figure 7 (Effect of 5 mM EGTA and 12.5 μM trifluoperazine dihydrochloride (TFP) on conidiation and H_2O_2 accumulation during conidiation of *Bgt*). Wheat leaf segments of Chancellor were inoculated with isolate 21-2. A pilot experiment with a series concentration of the two chemicals (dissolved in 0.025% Tween 20) was carried out by spraying at 1dpi and the haustorium formation rates were investigated at 3dpi (A). Based on the resulting data, 5 mM EGTA and 12.5 μM TFP were applied at 4 dpi by spraying the solutions. Infected leaf segments were sampled at 5dpi and 6dpi and stained with the typan blue and DAB solution. Then microscopy observation was carried out. Number of foot cells per single colonies at 5dpi (B), number of conidiophores per single colonies at 6dpi (C), conidia concentration of 2.5 mL conidiospores suspension dislodged from 2.5 cm long diseased leaf segments at 10dpi (D), mycelium expansion width of single colony at 100× magnification at 5dpi (E) and percentage of colony with DAB stained conidiophore at 5dpi (F) were investigated with three replicates. For each replicate, thirty colonies were observed. Values are means of data with three replicates of each treatment and error bars indicate standard error of the means. The significance of the differences between the treatments and untreated control was determined by Student's *t* tests. Significant decreases in the rate of foot cell and conidiophore formation and conidia production after 5 mM EGTA and 12.5 μM TFP treatment occurred ($p < 0.01$, indicated with two asterisks). The two treatments have significant impacts on both the mycelium growth and the rate of colonies stained with DAB ($p < 0.05$, indicated with one asterisk).
